# Lung-heart toxicity in a randomized clinical trial of hypofractionated image guided radiation therapy for breast cancer

**DOI:** 10.3389/fonc.2023.1211544

**Published:** 2023-11-20

**Authors:** Hilde Van Parijs, Elsa Cecilia-Joseph, Olena Gorobets, Guy Storme, Nele Adriaenssens, Benedicte Heyndrickx, Claire Verschraegen, Nam P. Nguyen, Mark De Ridder, Vincent Vinh-Hung

**Affiliations:** ^1^ Universitair Ziekenhuis Brussel, Vrije Universiteit Brussel, Brussels, Belgium; ^2^ Department of Oral Surgery, University Hospital of Martinique, Fort-de-France, France; ^3^ Department of Medical Oncology, The Ohio State University Comprehensive Cancer Center, Columbus, OH, United States; ^4^ Department of Radiation Oncology, Howard University, Washington, DC, United States; ^5^ Department of Clinical Research, International Geriatric Radiotherapy Group, Washington, DC, United States; ^6^ Department of Radiotherapy, Institut Bergonié, Bordeaux, France

**Keywords:** time-to-alteration analysis, breast neoplasms, targeted radiotherapy, pulmonary injury, heart damage, health-related quality of life

## Abstract

**Background:**

TomoBreast hypothesized that hypofractionated 15 fractions/3 weeks image-guided radiation therapy (H-IGRT) can reduce lung-heart toxicity, as compared with normofractionated 25-33 fractions/5-7 weeks conventional radiation therapy (CRT).

**Methods:**

In a single center 123 women with stage I-II operated breast cancer were randomized to receive CRT (N=64) or H-IGRT (N=59). The primary endpoint used a composite four-items measure of the time to 10% alteration in any of patient-reported outcomes, physician clinical evaluation, echocardiography or lung function tests, analyzed by intention-to-treat.

**Results:**

At 12 years median follow-up, overall and disease-free survivals between randomized arms were comparable, while survival time free from alteration significantly improved with H-IGRT which showed a gain of restricted mean survival time of 1.46 years over CRT, P=0.041.

**Discussion:**

The finding establishes TomoBreast as a proof-of-concept that hypofractionated image-guided radiation-therapy can improve the sparing of lung-heart function in breast cancer adjuvant therapy without loss in disease-free survival. Hypofractionation is advantageous, conditional on using an advanced radiation technique. Multicenter validation may be warranted.

**Trial registration:**

https://clinicaltrials.gov/ct2/show/NCT00459628. Registered 12 April 2007.

## 1 Introduction

Considerable evidence has accumulated that post-operative radiation therapy for breast cancer is an important adjuvant treatment to reduce the risk of local recurrence and the risk of breast cancer mortality. However, it has been long known that the improvement in overall survival is small. In a historical meta-analysis of 36 breast cancer trials, Van de Steene et al. observed in 2000 that adjuvant radiotherapy (using current techniques at that time, standard or “safe” fractionation) improved overall survival (1). They retained a chronological year breakpoint of 1970. The breakpoint coincided with trials using megavoltage equipment, simulation and computerized tomography planning prior to radiotherapy, resulting in differences in target volume coverage and reduced normal tissue toxicities, and subsequent odds reduction of mortality. In addition, the authors brought forward the idea that local-regional control and overall survival are two different end points in early breast cancer treatment, linked by local-regional relapse but separated by normal tissue toxicity and prognosis for patients. They stressed the importance of reducing cardiovascular and other types of late toxicity.

At the time of three meta-analyses on the subject (1–3), breast cancer radiotherapy was conducted mostly using static opposed tangential radiotherapy fields. Low priority was given to heart and lung doses. Target coverage generously included a quarter or more of the thoracic wall, from a beam’s entry point mid-sternal to the opposed tangential point at mid-axillary thickness. Constraint guidance was limited to a few geometrically specified measures. Portal images were acquired once at the first treatment session, and thereafter infrequently. Meanwhile, tomotherapy became available at the Universitair Ziekenhuis Brussel (UZ Brussel) in summer 2006 (4). Tomotherapy is an integrated intensity-modulated radiotherapy (IMRT) helical system that combines a rotational linear accelerator delivering beamlets and a translational couch technique with integrated megavolt computed tomography allowing volumetric image guidance radiotherapy (IGRT), with the facility of online daily 3-dimensional image matching and repositioning. The system was found to be workload intensive (5). As long as the benefit of breast radiotherapy was only in reducing the risk of recurrence, there was no reason to switch to a time-intensive technique which necessarily would reduce availability to other tumors. However, as evidence emerged that there was also a survival advantage linked to the technique (probably due to a reduction in toxicity), the determination of how to improve this advantage became a compelling question. It was clear that the routine techniques had to be reconsidered. In early evaluations, tomotherapy for breast cancer appeared promising (6). TomoBreast was designed to test the hypothesis that hypofractionated image-guided radiation therapy (H-IGRT) using tomotherapy can substantially reduce lung and heart toxicities, as compared with normofractionated conventional radiation therapy (CRT). Herein we report the end result of the trial, accordingly.

## 2 Methods

The primary endpoint was combined pulmonary and cardiac toxicities, as determined by medical imaging (abandoned for lack of funding) and functional assessments that included patient reported outcomes, physician clinical evaluation, pulmonary function tests, and heart echocardiography. The secondary endpoint was local-regional recurrences. The study population consisted of women presenting with histologically proven stage I or II (T1-3N0 or T1-2N1 M0) invasive breast carcinoma (7). Inclusion criteria were: informed consent, age ≥ 18 years, complete surgical resection, and pre-operative imaging with computed tomography, magnetic resonance imaging, and/or positron emission tomography scan. Patients were excluded if they had previously received radiotherapy to the breast or to other thoracic sites, or were pregnant, or breastfeeding, or were premenopausal without contraception, or presented with psychiatric or addictive disorders.

The trial was approved by the UZ-Brussel ethics committee and registered on ClinicalTrials.gov, NCT00459628. All participants gave written informed consent. They were randomized to either the CRT (control arm), or the H-IGRT (experimental arm).

CRT treatment planning used the Pinnacle3 planning system (ADAC-laboratories, Milpitas, CA, USA). Hypofractionation was not yet standard, hence the control arm used normofractionation. The prescribed dose was 50 Gy in 25 fractions over 5 weeks to the breast/chest wall by tangential opposed beams and in cases of nodal involvement to axillary/supraclavicular areas by an anterior field. Dose modulation was implemented as field-in-field compensation. A sequential electron boost delivered 16 Gy in 8 fractions over 2 weeks to the tumor bed in lumpectomy patients ≤70 years of age (8). The boost dose was calculated to a depth of 2-3 cm without individualized treatment plan. CRT dose constraints were not prespecified. Lung-in-field was accepted up to 3 cm central distance, and heart-in-field up to 2 cm. Breath hold techniques were not available in our center at that time.

For H-IGRT treatment planning, inverse-IMRT using the Tomotherapy Planning System (Accuray, Sunnyvale, CA, USA) was applied, for a prescription of 42 Gy in 15 x 2.8 Gy fractions over 3 weeks to the breast/chest wall and to nodal areas in case of positive lymph nodes, and a simultaneous integrated boost of 9 Gy in case of lumpectomy. H-IGRT procedure recommended dose-constraints to various organs and targets (**Supplementary Material**). Hypofractionation was chosen for the experimental arm due to the higher workload of tomotherapy (5). The schedule was designed on consideration of the Canadian breast trial (9), and on consideration of other hypofractionation protocols available in 2007, as detailed in the report of the pre-TomoBreast pilot study (10).

### 2.1 Statistical analyses

The trial required ≥118 patients on the hypothesis that any-grade lung-heart toxicity would be reduced from 25% with CRT (11, 12), to 5% with H-IGRT, by two-sided testing with a power of 0.80 at a significance level of 0.05. Randomization was balanced by nodal status, type of surgery, and chemotherapy sequence using Efron’s biased coin method (13). Eligibility was verified by a trial coordinator and patients were assigned after consent to a treatment arm by computer script of the method, independently of the physicians in charge of the patients.

Lung and heart were assessed prior to therapy, 1-3 months after therapy, and then once yearly. Data were abstracted from the patients’ medical charts, including: (i) pulmonary function tests: forced expiratory volume in 1 s (FEV1), forced vital capacity (FVC), diffusing capacity of the lungs for carbon monoxide (DLCO), alveolar volume at total lung capacity (VA), residual volume (RV), and total lung capacity (TLC) (14); (ii) echocardiography left ventricular ejection fraction (LVEF) (15); (iii) clinical toxicity grading using the Radiation Therapy Oncology Group (RTOG) late radiation morbidity grades for lung and heart (16); and (iv) patient’s self-reported symptoms of fatigue and dyspnea abstracted from the European Organization for Research and Treatment of Cancer (EORTC) Quality of Life questionnaire QLQ-C30 (17). For the pulmonary function tests and LVEF, raw measurements were used, with predicted values discarded to avoid spurious effects caused by changes of reference (18). RV was inverted as RVinv to maintain consistency with the other pulmonary functions in which decreasing values indicate declining function. Data on cosmesis, shoulder-arm morbidity, and items other than fatigue and dyspnea were collected but are not considered in the present report.

Time measurements used the date of randomization as the origin. Toxicities over time were evaluated on an ordinal scale and on a continuous scale.

On an ordinal scale provided for descriptive purpose, the lung function tests and the heart’s LVEF were categorized using cutoffs adapted from the Common Terminology Criteria for Adverse Events (CTCAE v.3), which classified the grades by the percent change from predicted for FEV1, and by the absolute change for resting ejection fraction (19). Specifically, for the lung function tests, we mapped the percent change from baseline of (10%,25%] to grade 1, (25%,50%] to grade 2, (50%,75%] to grade 3, and >75% to grade 4. For LVEF, we mapped the change from baseline of (10%,20%], (20%,30%], (30%,50%], and >50% onto LVEF grades 1, 2, 3, and 4, respectively. The RTOG grades were used as-is. The QLQ-C30 fatigue and dyspnea symptom were scaled to 0-100 and then categorized into [0,20] mild, (20,40] moderate and >40 severe – there is no clear recommendation for categorizing the QLQ, we derived the latter cutoff from a review of fatigue (20). The grades were summarized by simple tabulation of all observations. Tests for proportions were not retained to avoid confusing descriptive categorization with the inferential purpose of the study, as detailed below.

On the continuous scale, which was retained for inference on the trial outcome, a “time to 10% alteration” (TTA) was defined as the time for a lung or a heart indicator to reach a d% = 10% deterioration. Generalizing on (21) to any measurement, the TTA was computed for each patient using the least square regression fit to each of the patient’s sets of time measurements. If the regression showed no significant decline in any of the measurements, or if the computed TTA exceeded the patient’s time to follow-up, the patient was censored for toxicity at the patient’s actual follow-up time. A composite toxicity event was defined as the first occurrence of any of the following: lung TTA, heart TTA, RTOG grade increase or fatigue-dyspnea increase. The Kaplan-Meier method (22), the restricted mean survival time (23) and the log-rank test (24) were used to analyze the composite TTA.

Subgroup analyses considered age, weight, tumor laterality, type of surgery, radiation to regional lymph node areas, chemotherapy, trastuzumab therapy, and histological grade interaction with the randomization arm in a Cox proportional hazard model of the TTA. Management of missing data and partial imputation of LVEF were done in an earlier preprint at 5 years. Later updated data retrieval, corrections and interpolation between medical patient contacts until and after the Covid pandemic have been reported recently (25).

All analyses were done by intent-to-treat without any exclusion. The computations used R version 4.1.2 (26). Implementation packages were: “arsenal” for tables, with the default Student’s t-test for the comparison of means and Chi-square test with continuity correction for the comparison of proportions; “survival” for Kaplan-Meier time-to-event analysis, log-rank test, restricted mean survival times [with “survRM2” (27)], and Cox models (28); “Amelia” for multiple time series imputation in an interim analysis (29); “forestplot” for subgroup results (30). Computation of TTA used R and “survival” built-in functions.

## 3 Results

**Figure 1:**
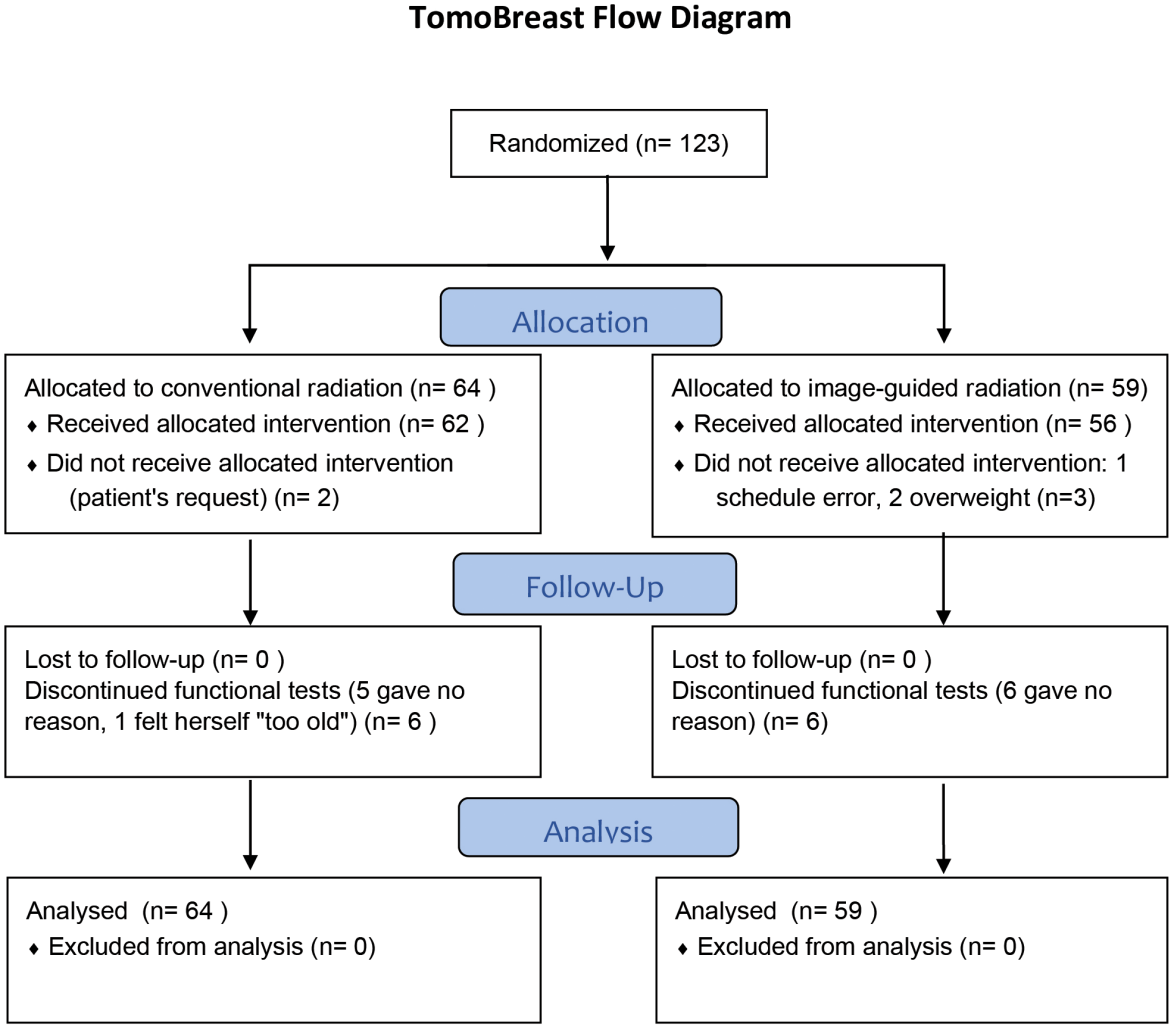
CONSORT Diagram. Reproduced from BMC Cancer 2021, Hilde Van Parijs et al, shared through https://rdcu.be/dqGVy under a Creative Commons Attribution 4.0 International License.

The trial started in May 2007 and ended accrual in July 2011. A total of 123 women consented to participate. Of these, 64 were randomized to CRT and 59 to hypofractionated image guided radiation therapy (H-IGRT). Of the 64 patients allocated to CRT, 2 received H-IGRT by request. Of the 59 patients allocated to H-IGRT, three received CRT; one because of an appointment scheduling error, two because tomotherapy was unsuitable due to the patient’s body size exceeding the system’s limits (**Figure 1**). As of July 16th, 2021, the median follow-up of the 103 surviving was 12.0 years (range 10.0 to 14.0 years) from randomization.

The patients’ characteristics were well balanced between the two arms, although there were exceptions, notably a higher frequency of axillary lymph node dissection (P = 0.043) and HER2 overexpression (P = 0.055) in the H-IGRT patients (**Table 1**). H-IGRT patients were 2 years younger than CRT patients, but the difference did not reach significance. Hormone therapy matched patient’s age and receptor status, but for one patient in whom both receptors were coded negative who received letrozole, and two patients coded as estrogen receptor (ER) positive who received no hormone therapy. Genomic assay of breast cancer and Ki-67 were not available. Nevertheless, surrogate molecular subtyping (31, 32) showed no imbalance other than the HER2 overexpression (**Table 1**). Regarding cardiovascular comorbidities, arterial hypertension alone was recorded in 17 patients (=13.8% of 123), representing the most prevalent cardiovascular pathology. Arterial hypertension associated with another cardiovascular condition (coronary disease, valvular or vascular pathology) was recorded in 5 patients (=4.1% of 123). Cardiovascular pathology (vascular, valvular and/or arrhythmia) without report of arterial hypertension was recorded in 7 patients (=5.7% of 123). The distribution of cardiovascular disease was comparable between the two randomization arms (**Table 1**). Regarding respiratory disease, 5 patients (= 4.1% of 123) were recorded as presenting with a history of asthma without other specification, and 2 (= 1.6% of 123) as presenting with a history of respiratory condition (1 chronic bronchitis, 1 mild pulmonary hypertension). The distribution of respiratory disease was comparable between the two randomization arms (**Table 1**).

**Table 1:** Patients’ characteristics.

Characteristic	Unit or Level	CRT (N=64)	H-IGRT (N=59)	p
Age at randomization: mean (sd)	Years	57.8 (11.6)	55.1 (11.5)	0.198
median (range)	Years	54.8 (35.7, 81.0)	52.7 (31.7, 80.0)	
Karnofsky Performance Status: mean (sd)	%	94.1 (8.4)	94.7 (7.2)	0.678
	N Missing	1	4	
Weight: mean (sd)	*kg*	67.0 (12.3)	69.5 (15.4)	0.335
Height: mean (sd)	*cm*	161.5 (6.7)	163.4 (6.5)	0.118
Body Mass Index: mean (sd)	*kg/m2*	25.7 (4.2)	26.0 (5.4)	0.720
Smoker status: N (%)	No	46 (71.9%)	38 (64.4%)	0.493
	Yes	8 (12.5%)	12 (20.3%)	
	Former	10 (15.6%)	9 (15.3%)	
Smoking quantity: mean (sd)	Packyear	6.8 (14.3)	7.1 (14.2)	0.925
	N missing	2	1	
History of cardiovascular disease: N (%)	None reported	50 (78.1%)	44 (74.6%)	0.328
	Arterial hypertension (AHT)	7 (10.9%)	10 (16.9%)	
	AHT + vascular	0 (0.0%)	2 (3.4%)	
	AHT + valvular	1 (1.6%)	0 (0.0%)	
	AHT + coronary	2 (3.1%)	0 (0.0%)	
	Vascular	1 (1.6%)	2 (3.4%)	
	Valvular + arrythmia	1 (1.6%)	1 (1.7%)	
	Arrythmia	2 (3.1%)	0 (0.0%)	
History of respiratory disease: N (%)	None reported	60 (93.8%)	56 (94.9%)	0.935
	Asthma	3 (4.7%)	2 (3.4%)	
	Other	1 (1.6%)	1 (1.7%)	
Mastectomy: N (%)	Yes	19 (29.7%)	26 (44.1%)	0.142
Axillary Dissection: N (%)	Yes	19 (29.7%)	29 (49.2%)	0.043
Chemotherapy: N (%)	No	38 (59.4%)	29 (49.2%)	0.499
	Before RT	7 (10.9%)	7 (11.9%)	
	Concomitant RT	19 (29.7%)	23 (39.0%)	
Hormone therapy: N (%)	No	9 (14.1%)	8 (13.6%)	0.155
	Tamoxifen	26 (40.6%)	16 (27.1%)	
	Aromatase inhibitor	26 (40.6%)	26 (44.1%)	
	LHRH agonist	3 ( 4.7%)	9 (15.3%)	
Trastuzumab therapy (HER2+): N (%)	Yes	3 ( 4.7%)	10 (16.9%)	0.055
Nodal irradiation: N (%)	Yes	16 (25.0%)	20 (33.9%)	0.376
Node-positive: N (%)		16 (25.0%)	21 (35.6%)	0.279
Laterality: N (%)	Right	31 (48.4%)	24 (40.7%)	0.402
	Left	32 (50.0%)	35 (59.3%)	
	Bilateral	1 ( 1.6%)	0 ( 0.0%)	
Histological Grade: N (%)	1	18 (30.0%)	16 (28.1%)	0.549
	2	25 (41.7%)	29 (50.9%)	
	3	17 (28.3%)	12 (21.1%)	
	N Missing	4	2	
Stage: N (%)	I	28 (43.8%)	25 (42.4%)	0.577
	IIA	31 (48.4%)	26 (44.1%)	
	IIB	5 ( 7.8%)	8 (13.6%)	
Tumor Size Pooled: mean (sd)	*mm*	19.8 (11.0)	20.3 (11.6)	0.820
Estrogen receptor (ER) positive: N (%)	Yes	56 (87.5%)	48 (81.4%)	0.489
Progesterone receptor (PR) positive: N (%)	Yes	46 (71.9%)	46 (78.0%)	0.569
Molecular subtype surrogates: N (%)	Luminal A, ER+ HER2– Grade 1-2	40 (67.8%)	34 (61.8%)	0.157
	Luminal B, ER+ HER2– Grade 3	10 (16.9%)	6 (10.9%)	
	HER2+	3 (5.1%)	10 (18.2%)	
	Triple negative, ER– PR– HER2–	6 (10.2%)	5 (9.1%)	
	N Missing	5	4	

RT, radiotherapy; sd, standard deviation.

Regarding baseline measurements, H-IGRT patients had a better LVEF as a continuous measure (not as an ordinal one), and a better FEV1, which was cancelled when entered into the ratio FEV1/FVC, P = 0.321 (**Table 2**).

**Table 2:** Baseline measurements.

Measurement	CRT (n=64)	H-IGRT (n=59)	p
FEV1 Liters, mean (SD)	2.44 (0.56)	2.65 (0.59)	0.041
FVC Liters, mean (SD)	3.26 (0.67)	3.47 (0.67)	0.084
FEV1/FVC ratio, mean (SD)	0.75 (0.07)	0.76 (0.07)	0.321
DLCO mL/mmHg/min, mean (SD)	18.74 (3.98)	18.97 (3.42)	0.611
VA Liters, mean (SD)	4.46 (0.68)	4.62 (0.68)	0.177
DLCO/VA ratio, mean (SD)	4.20 (0.67)	4.12 (0.57)	0.553
RV Liters, mean (SD)	1.90 (0.51)	1.85 (0.51)	0.637
TLC Liters, mean (SD)	5.20 (0.70)	5.36 (0.78)	0.258
LVEF % ordinal			0.270
50-60% (%)	15 (26.8)	9 (16.4)	
>60% (%)	41 (73.2)	46 (83.6)	
LVEF % continuous, mean (SD)	62.62 (4.54)	64.82 (5.92)	0.047
Fatigue raw score, mean (SD)	1.90 (0.62)	2.06 (0.75)	0.207
Dyspnea raw score, mean (SD)	1.33 (0.67)	1.46 (0.79)	0.352

FEV1, forced expiratory volume in 1 s; FVC, forced vital capacity; DLCO, diffusing or transfer capacity of the lungs for carbon monoxide; VA, alveolar volume at total lung capacity; RV, residual volume; TLC, total lung capacity; LVEF, left ventricular ejection fraction.

**Figure 2:**
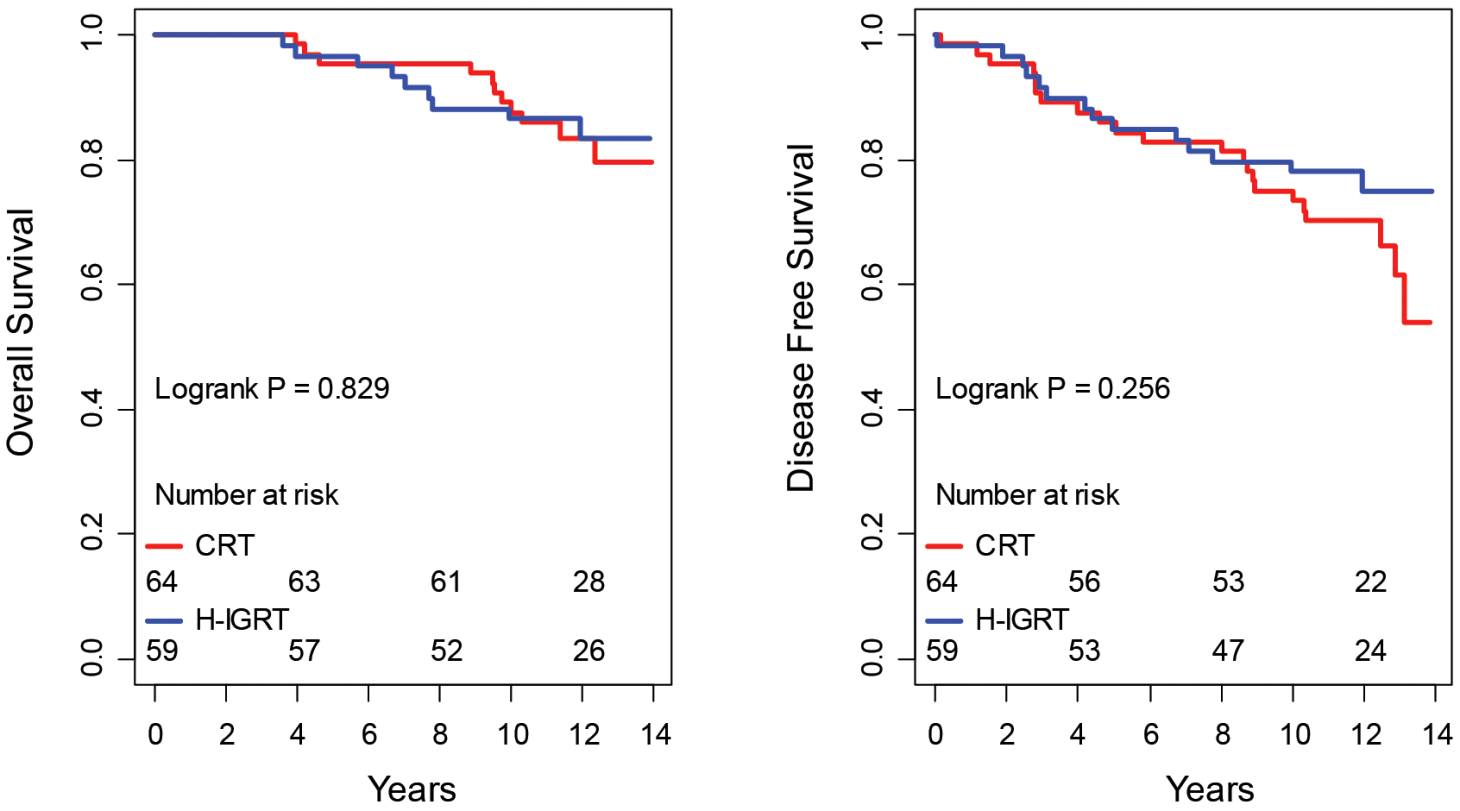
Overall and disease-free survival.

CRT and H-IGRT did not differ significantly either by overall survival (event: death from any cause) or by disease-free survival (event: any of death or first occurrence of local or regional recurrence, metastasis, or new primary), P = 0.829 and P=0.256, respectively (**Figure 2**). The estimated 10-year overall survival rates were 89.1% in the CRT arm and 86.4% in the H-IGRT arm. The restricted mean overall survival time in the CRT *vs*. H-IGRT arm at a horizon of 13.5 years (near the common maximal observation in the two arms) were 12.6 *vs*. 12.5 years. The estimated 10-year disease-free survival rates were 75.0% in the CRT arm and 78.0% in the H-IGRT arm. The restricted mean disease-free survival time in the CRT *vs*. H-IGRT arm at a horizon of 13.5 year was 11.1 *vs*. 11.5 years. Of note regarding new primaries, contralateral breast cancer was observed in one H-IGRT patient (invasive), *vs*. three CRT patients (2 invasive, 1 in situ), and one lung cancer was observed in each of the H-IGRT and CRT arm.

On an ordinal-categorical scale, there were few Grade 3 or 4 toxicity (**Table 3**). Absence of toxicity (grade 0) was preponderant with H-IGRT in most evaluations, FEV1, FVC, DLCO, VA, TLC, RTOG Lung, RTOG Heart and Dyspnea, but not in RVinv, LVEF and Fatigue. Considering for example the item Dyspnea at Follow-up >5 years, there were 270 + 159 + 58 = 487 Dyspnea measurements. Moderate to Severe Dyspnea represented 32.6%+11.9% = 44.5% of these measurements, versus 55.4% Mild Dyspnea for CRT, meaning that the odds of Moderate-Severe Dyspnea versus Mild Dyspnea were 44.5/55.4 = 0.803 with CRT, as compared with the reduced odds of (24.1 + 13.5 = 37.6)/62.4 = 0.603 with H-IGRT. That is, Dyspnea was in favor of H-IGRT. Conversely, considering the item Fatigue at Follow-up > 5 years, the odds of Moderate-Severe versus Mild Fatigue were (34.7 + 25.9 = 60.6)/39.4 = 1.538 with CRT, as compared with the increased odds of Fatigue (41.3 + 32.7 = 74.0)/26.0 = 2.846 with H-IGRT. That is, Fatigue was in favor of CRT. Clearly, interpretation of the categorized results is not straightforward, although useful to provide at a glance the number of measurements available. The ordinal categorization was not the mainstay for inference, P-values were not computed.

**Table 3:** Ordinal deterioration and toxicity grades relative to baseline.

Grade	Follow-up ≤ 5years		Follow-up > 5 years	
	CRT N=1571	H-IGRT N=1462	CRT N=893	H-IGRT N=721
FEV1				
0	887 (76.7%)	948 (78.2%)	92 (27.4%)	104 (34.2%)
1	259 (22.4%)	252 (20.8%)	180 (53.6%)	180 (59.2%)
2	10 (0.9%)	12 (1.0%)	59 (17.6%)	20 (6.6%)
3	0 (0.0%)	0 (0.0%)	5 (1.5%)	0 (0.0%)
FVC				
0	888 (76.8%)	947 (78.1%)	88 (26.2%)	123 (40.5%)
1	260 (22.5%)	261 (21.5%)	173 (51.5%)	153 (50.3%)
2	8 (0.7%)	4 (0.3%)	69 (20.5%)	28 (9.2%)
3	0 (0.0%)	0 (0.0%)	6 (1.8%)	0 (0.0%)
DLCO				
0	712 (62.2%)	858 (71.8%)	144 (45.1%)	192 (71.1%)
1	421 (36.8%)	315 (26.4%)	117 (36.7%)	60 (22.2%)
2	12 (1.0%)	22 (1.8%)	44 (13.8%)	12 (4.4%)
3	0 (0.0%)	0 (0.0%)	14 (4.4%)	6 (2.2%)
VA				
0	943 (82.4%)	1040 (87.0%)	216 (71.1%)	221 (81.9%)
1	202 (17.6%)	150 (12.6%)	81 (26.6%)	37 (13.7%)
2	0 (0.0%)	5 (0.4%)	7 (2.3%)	12 (4.4%)
RVinv				
0	879 (77.9%)	868 (72.3%)	175 (57.4%)	73 (28.5%)
1	198 (17.6%)	240 (20.0%)	62 (20.3%)	93 (36.3%)
2	51 (4.5%)	85 (7.1%)	68 (22.3%)	77 (30.1%)
3	0 (0.0%)	7 (0.6%)	0 (0.0%)	13 (5.1%)
TLC				
0	872 (77.3%)	999 (83.2%)	187 (58.4%)	222 (86.7%)
1	249 (22.1%)	198 (16.5%)	119 (37.2%)	34 (13.3%)
2	7 (0.6%)	3 (0.2%)	14 (4.4%)	0 (0.0%)
LVEF				
0	1171 (96.2%)	1012 (92.5%)	329 (87.0%)	174 (85.3%)
1	43 (3.5%)	81 (7.4%)	37 (9.8%)	28 (13.7%)
2	3 (0.2%)	1 (0.1%)	10 (2.6%)	2 (1.0%)
3	0 (0.0%)	0 (0.0%)	2 (0.5%)	0 (0.0%)
RTOG Lung				
0	1063 (76.8%)	983 (80.5%)	548 (79.1%)	498 (84.6%)
1	228 (16.5%)	191 (15.6%)	49 (7.1%)	21 (3.6%)
2	73 (5.3%)	42 (3.4%)	68 (9.8%)	32 (5.4%)
3	21 (1.5%)	5 (0.4%)	19 (2.7%)	36 (6.1%)
4	0 (0.0%)	0 (0.0%)	9 (1.3%)	2 (0.3%)
RTOG Heart				
0	1303 (93.3%)	1229 (94.4%)	576 (80.4%)	509 (85.5%)
1	58 (4.2%)	40 (3.1%)	35 (4.9%)	26 (4.4%)
2	25 (1.8%)	32 (2.5%)	59 (8.2%)	50 (8.4%)
3	9 (0.6%)	1 (0.1%)	34 (4.7%)	6 (1.0%)
4	2 (0.1%)	0 (0.0%)	12 (1.7%)	4 (0.7%)
Fatigue scale (0-100)				
Mild [0,20]	404 (30.0%)	285 (21.6%)	192 (39.4%)	106 (26.0%)
Moderate (20,40]	568 (42.2%)	620 (47.1%)	169 (34.7%)	168 (41.3%)
Severe (40,100]	375 (27.8%)	412 (31.3%)	126 (25.9%)	133 (32.7%)
Dyspnea scale (0-100)				
Mild [0,20]	772 (57.3%)	774 (58.8%)	270 (55.4%)	254 (62.4%)
Moderate (20,40]	453 (33.6%)	362 (27.5%)	159 (32.6%)	98 (24.1%)
Severe (40,100]	122 (9.1%)	181 (13.7%)	58 (11.9%)	55 (13.5%)

FEV1, forced expiratory volume in 1 s; FVC, forced vital capacity; DLCO, diffusing or transfer capacity of the lungs for carbon monoxide; VA, alveolar volume at total lung capacity; RVinv, log(1/residual volume); TLC, total lung capacity; LVEF, left ventricular ejection fraction; RTOG, Radiation Therapy Oncology Group; N and figures below, number of measurement time points (% are column wise within each category of measurement).

**Figure 3:**
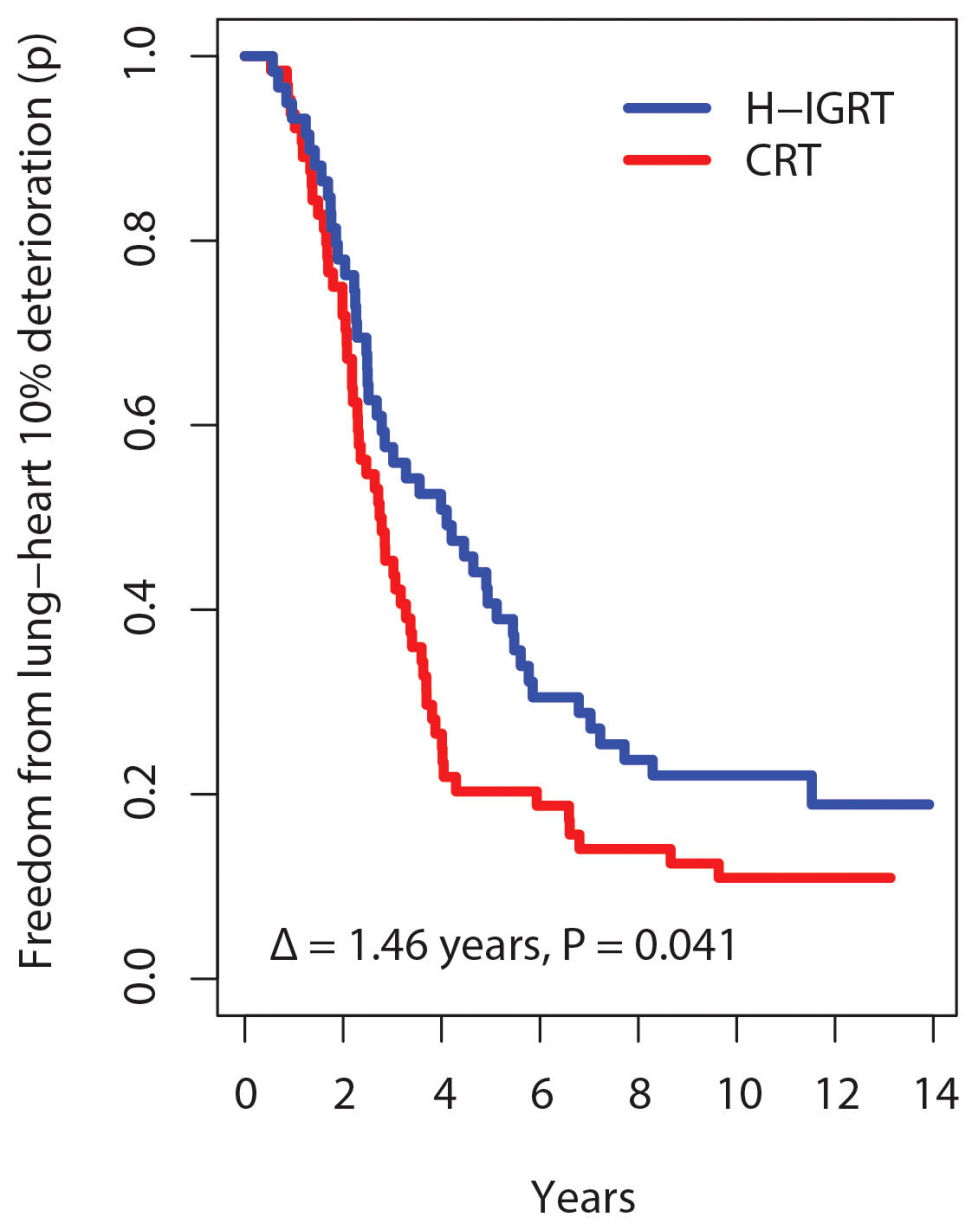
Kaplan-Meier probability (p) of freedom from 10% lung-heart deterioration. Δ, difference in restricted mean survival time at 13 years horizon.

On a continuous scale using a composite outcome that combined lung tests (FEV1/FVC, DLCO/VA, TLC), echocardiography (LVEF), clinical RTOG grades, and patient’s self-assessed fatigue-dyspnea scores over time, survival free from composite lung-heart alteration was significantly improved with H-IGRT, as shown in all measures of survival. The comparison of the survival curves displayed a wide difference, log-rank P=0.039. Estimates of freedom from alteration with H-IGRT *vs*. CRT were 40.7% *vs*. 20.3% at 5 years, and 22.0% *vs*. 10.9% at 10 years, respectively. The median time free from alteration was 4.10 years with H-IGRT, *vs*. 2.76 years with CRT. The expectancy (restricted mean) of time free from alteration at a horizon of 13 years (near the maximal observation time common to the two arms) was 5.50 years with H-IGRT, *vs*. 4.04 years with CRT, i.e. a difference Δ of 1.46 years, P=0.041 (**Figure 3**). By Cox analysis of the time to alteration, the H-IGRT hazard ratio was 0.67 (95% CI: 0.45–0.98), P=0.040, corresponding to a statistically significant 33% proportional reduction of composite toxicity.

**Figure 4:**
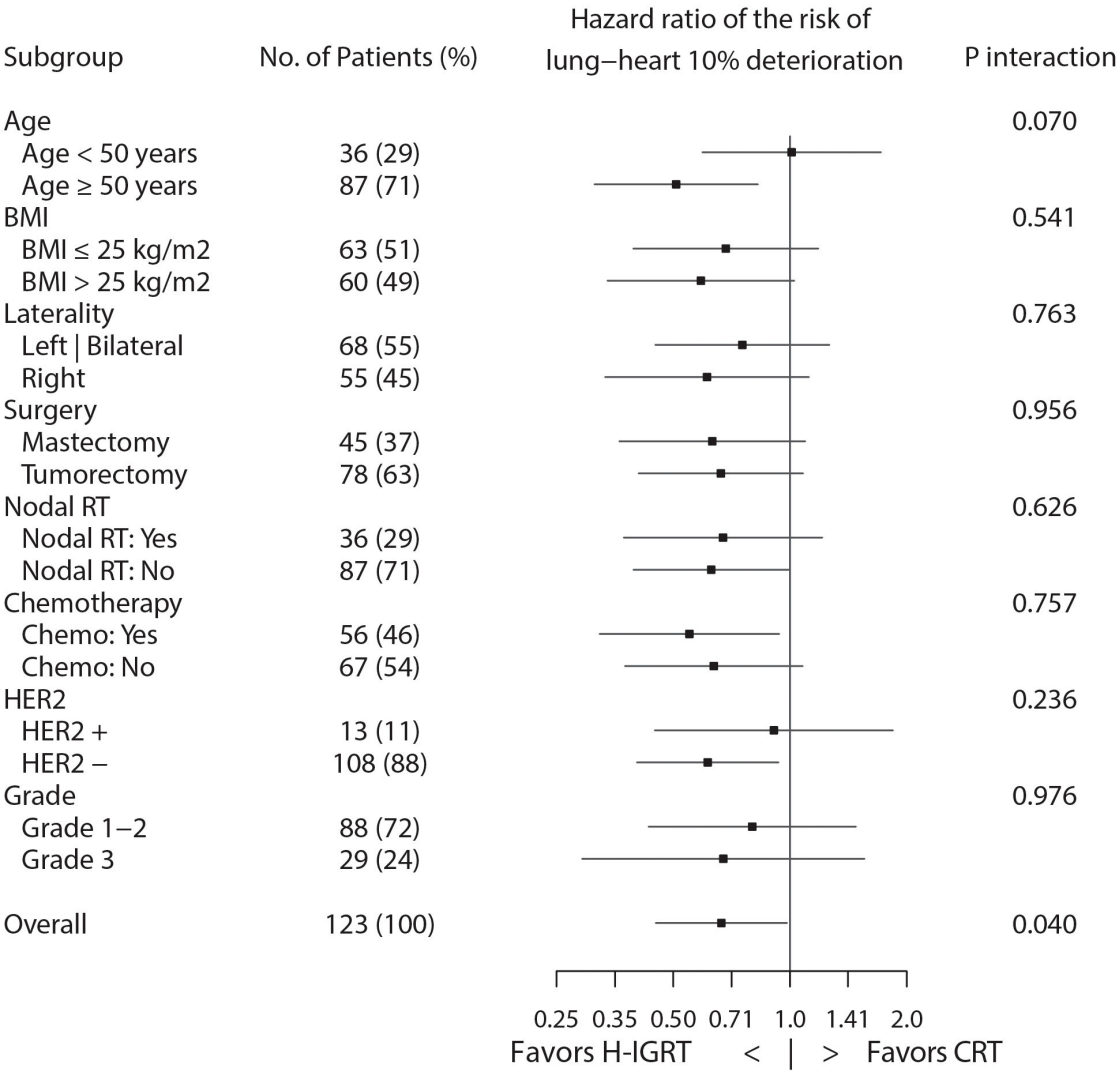
Forest plot of hazard ratios of lung-heart composite toxicity.

*Post-hoc* subgroup analysis of the time from alteration by Cox regression found no significant interaction, except age group approaching significance, P=0.070 (**Figure 4**). The forest plot suggested that patients ≥ 50 years derived a clear benefit from H-IGRT, but not so in patients < 50 years.

## 4 Discussion

### 4.1 Outcomes

Toxicity is a complex multi-dimensional concept. RTOG scoring combines several types of measures, patient’s symptoms, clinical findings, functional measures and imaging. The difficulty is in the reproducibility, representativity and interpretability. A result such as **Table 3** is practically unmanageable, requires repeated assessments of distinct endpoints, sometimes yielding contradictory results, as mentioned in Results regarding LVEF, Dyspnea or Fatigue. In the present study, we used the endpoint of time free from event, where event was defined as the “deterioration” in any of:

- patient reported outcome (PRO),
- RTOG toxicity score,
- left ventricular ejection fraction (LVEF),
- and pulmonary function test (PFT).

That is to say:

- if a patient had no deterioration in any of the above items, she had a survival free from cardio-pulmonary toxicity during her full follow-up.

- If a patient had a deterioration in any of the above items, she had a survival free from toxicity, up to the time a deterioration is detected, at a threshold of 10% further discussed below.

Thus, the comparison is not between any single indicators, but between survival times free from deterioration in any of the PRO, RTOG, LVEF or PFT measures.

H-IGRT significantly improved the expectancy of time free from deterioration by 1.46 years (**Figure 3**), without differences in overall or disease-free survival (**Figure 2**). How does the result compare with the literature? To our knowledge, this study is unique in analyzing simultaneously lung and heart outcomes by integrating the different classes of measurements – patient reported, clinician assessed, and functional exams. Studies that randomized patients between different radiotherapy techniques with the objective of assessing heart or lung function have been scarce, let alone with a large number of patients or with a long follow-up. Out of two randomized trials with published results, one assessed myocardial perfusion at 6 months in 57 patients with or without active breath control (33). The study was inconclusive to demonstrate a difference with either technique. In the other published randomized study, pulmonary and cardiac perfusion and function were assessed at 1 year in 54 patients assigned to either IMRT with active breath hold or CRT in free breathing (34). Despite failing to show differences in lung per-fusion or lung function or in left anterior descending coronary perfusion defects, the study reported significantly less decline in LVEF with IMRT-breath hold.

Thus, while awaiting results of on-going trials such as the HARVEST hypofractionation with IMRT *vs*. normofractionation trial which will include echocardiography up to 5 years (35), TomoBreast adds twice as many patients and twelve years more follow-up to the published evidence that radiotherapy technique has a substantial impact to reduce lung and heart toxicity.

### 4.2 Hypofractionation

How could we attribute the improved lung-heart outcomes to H-IGRT, when the result could also have been due to hypofractionation? Attributing the present result to hypofractionation instead of the radiotherapy technique would have to assume that hypofractionation is an independent favorable factor with an effect size on lung and heart sufficiently large to account for the 33% proportional reduction of the risk of lung-heart toxicity, or large enough to improve the expectancy of time free of deterioration by 1.46 years, or enough to improve the 5-year freedom from deterioration, from 20.3% with CRT to 40.7% with H-IGRT?

In an on-going review of randomized clinical trials of hypofractionation in breast cancer, selected trials were those with 1) a conventional fractionation arm in order to have a common denominator; 2) hypofractionation with the same technique and same target volumes as conventional fractionation arm; 3) with a minimum follow-up of 2 years to allow for putative complete recovery after any potential adjuvant chemotherapy or any year-long trastuzumab; and 4) reporting a lung or heart outcome. Out of 22 trials, 6 had lung outcome data, 9 had heart outcome data. There was no evidence of a reduction of lung or heart toxicity independently attributable to hypofractionation. If there is an effect, it was too small to be determined out of 6353 patients in the HF arms and 5370 patients in the conventional fractionation arms.

### 4.3 Degradation threshold

The present study used a threshold of 10% as a criteria to define deterioration, on consideration of the minimally important difference in quality of life studies, the precision of exams (36), and on consideration of thresholds for reporting toxicity in quantal data (37). This might be excessively low. On a 0-5 toxicity grade scale, 10% maps to “Grade 0.5”, that is a Grade < 1, not clinically meaningful. The 10% threshold was useful in a 5-years follow-up study for which we wanted the most sensitivity to detect early changes. We did not realize that with more than twice longer follow-up, age of patients increased substantially, the general health would decline, more and more symptoms and signs would be recorded when using a large panel of outcome indicators.

**Figure 5:**
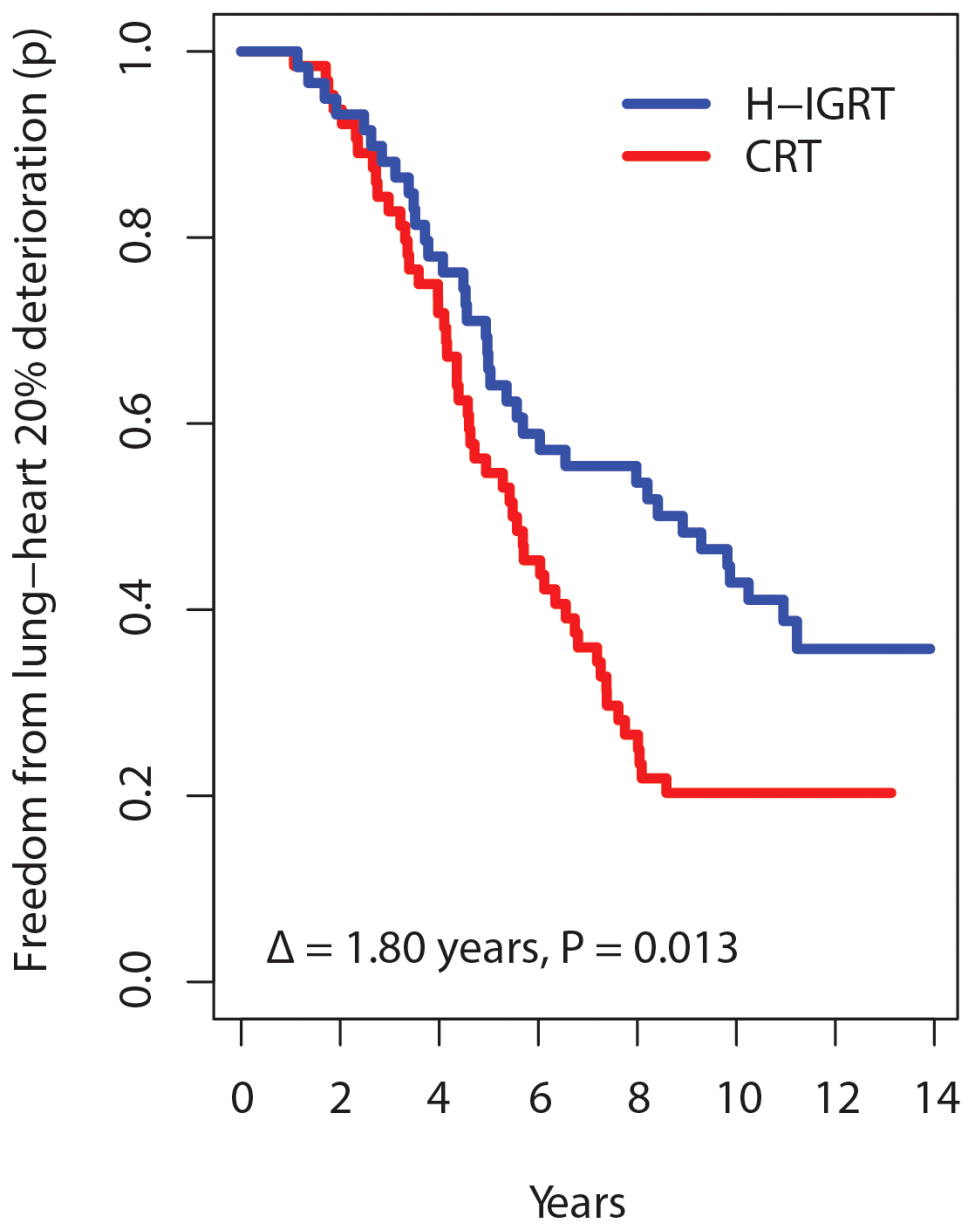
Survival time free from alteration according to a deterioration threshold of 20% instead of 10%.

What if different thresholds had been used in the present study? A d% of 20% maps into Grade 1, 40% into Grade 2, etc. With d% = 20%, the separation between the times free from deterioration increases to Δ = 1.80 years, P = 0.013 (**Figure 5**). With d% = 40%, the graph separation remains clear, Δ = 0.92 years, P = 0.116. With d% ≥60%, there were no separations and no significant differences. Thus, defining a d% value affects the chance of detecting a difference. With a small d%, there are too many events. But with a high d%, there were few TTA events, in keeping with the very low rates of severe toxicity, regardless of the randomization arm (**Table 3**). The pattern of the Δ’s as a function of d% suggests that the choice of H-IGRT at a follow-up of 12-13 years would matter mostly for Grade 1-2 toxicity.

Incidentally, with d% set at 20%, the TTA hazard ratio for H-IGRT over CRT was 0.59 (CI: 0.39-0.91), P=0.018, and the suggestion of a subgroup age effect (**Figure 4**) was not confirmed, P=0.111.

### 4.4 Quality of life improvement with image-guided radiotherapy

Our study highlights the importance of cardiac and lung sparing with advanced radiotherapy techniques to preserve patient quality of life. Indeed, the tomotherapy arm patients experienced significant improvement of dyspnea and fatigue (36). Pulmonary function test deterioration has been reported following adjuvant chemotherapy and radiotherapy for breast cancer and was considered as a tradeoff for increased relapsed free survival with the conventional radiotherapy technique (38). In one study, almost one-fifth of breast cancer patients developed clinical radiation pneumonitis associated with a significant decline in all pulmonary function parameters (39). Reduction of pulmonary function is correlated with the ipsilateral lung volume irradiated (V20>30%) which could be reduced with Tomotherapy constraint (40). To date, our study is the first to demonstrate a significant improvement in quality of life of breast cancer patients in parallel with the preservation of pulmonary function. As older women are at higher risk for cardiopulmonary complications due to preexisting comorbidities, hypofractionation radiotherapy with modern radiotherapy techniques such as Tomotherapy would be cost effective for elderly breast cancer patients.

### 4.5 Limitations and strengths

The know-how for time to alteration analysis was acquired late after trial completion. The time to alteration can be affected by the choice of the outcomes to be combined, by the type of regression implemented, and by the threshold of alteration as shown in the preceding paragraph. An ideal trial would have to specify in advance what parameters were selected. Much research needs to be done, such as identifying if weights need to be applied to the outcomes or not (41). Medical imaging follow-up could not be implemented. Prone planning was considered but was not sustained during the trial. Prone breast radiotherapy has emerged as a potential highly cost-effective technique to spare lung and heart (42, 43). Few patients received the double simulation, which with hindsight should have been offered to CRT patients too. At the time of the trial, breath-hold was not yet available in our center. Breath-hold has been shown to reduce the delivered dose to the heart (44). Patients and clinicians were not blinded to treatment allocation, crossovers occurred although these did not appear to affect the result. TomoBreast trial size was not designed to study subgroups, and neither was it designed to investigate recurring questions such as the risk of secondary tumors whenever a study of IMRT is done. No unusual occurrence of new primaries was observed, but the question can only be addressed in large trials. The population of randomized patients was heterogeneous and was limited to a single center. There is no guarantee that results would apply in other environments, external validation is needed.

We used LVEF as a distinct measurement, considering that ejection fraction is the standard to assess onco-cardiac toxicity. But other measures were also taken into account for RTOG scoring, with notably assessment of electrocardiographic anomalies such as T wave changes, arrhythmia, and non-echocardiographic imaging such as cardiac enlargement. The difference was that most LVEF were assessed with or without symptoms, whereas electrocardiography was done on demand according to patient’s clinical status.

H-IGRT was implemented with the tomotherapy system. Arguably comparable or even better dose distribution might be obtained with other techniques. Modern breast radiotherapy has evolved and improved a lot. Several techniques are available that can deliver the combination of highly modulated dose distributions with the possibility of frequent imaging. However, comparative clinical trials reporting on lung-heart toxicity are too scarce to establish the superiority of any technique. The reduced toxicity is possibly due to better patient positioning with daily imaging on tomotherapy, allowing smaller safety margins. Another gain lays in the better dose distribution, delivering a more homogeneous dose to the breast, but less to the surrounding tissues. Other techniques reviewed in recent issues of the journal, such as prone positioning, breath hold, particles for various indications (45–48), can reduce the dose to the heart and other organs too. The present study provides sustained long term information that will be useful for future studies to identify the most cost-effective way to reduce lung-heart doses, and to evaluate alternative treatment paradigms such as partial breast irradiation or intraoperative radiation therapy. TomoBreast showed, in a randomized setting, that low-grade toxicity could be detected and significantly reduced as early as 5 years with a difference sustained up to 12-14 years. The same short course fractionation schedule appears feasible, with or without chemotherapy, boost, or nodal irradiation. Patients were managed by the same team, within a normal workflow. The non-restrictive selection criteria indicated a good likelihood of translating the trial’s experience to daily practice. The large number of lung and heart function assessments over time provides a large body of quantitative data that will contribute to knowledge in the management of breast cancer.

## 5 Conclusion

Image-guided radiation therapy improved the combined lung-heart outcomes, preserving cardiac-pulmonary function, reducing clinical toxicity and maintaining patient’s quality of life, with the added advantage of a more convenient short fractionation schedule. The TomoBreast trial is a proof-of-concept that advanced radiation techniques might be considered in the adjuvant therapy of breast cancer.

## Data availability statement

The datasets presented in this study can be found in online repositories. The names of the repository/repositories and accession number(s) can be found below: https://dx.doi.org/10.5281/zenodo.5919956.

## Ethics statement

The studies involving humans were approved by The Institutional Review Board (Ethics Committee) of the Universitair Ziekenhuis Brussel. The studies were conducted in accordance with the local legislation and institutional requirements. The participants provided their written informed consent to participate in this study.

## Author contributions

Concept/design: GS and VV-H. Provision of study materials or patients: HP, NA, BH, MR, and VV-H. Collection and/or assembly of data: HP, BH, MR, and VV-H. Data analysis and interpretation: EC-J, OG, NN, and VV-H. Manuscript original drafts: HP, GS, CV, NN, and VV-H. All authors contributed to the article and approved the submitted version.
