# Supplementary material for: Lung-heart toxicity in a randomized clinical trial of hypofractionated image guided radiation therapy for breast cancer
Source: Front Oncol. 2023 Nov 20;13:1211544. doi: 10.3389/fonc.2023.1211544 (PMC10694354; doi:10.3389/fonc.2023.1211544)
Supplement: Supplementary file 1 [file DataSheet_1.pdf]

frontiers

Supplementary Material

Lung-heart toxicity in a randomized clinical trial of hypofractionated image guided radiation therapy for breast cancer

Hilde Van Parijs, Elsa Cecilia-Joseph, Olena Gorobets, Guy Storme, Nele Adriaenssens, Benedicte Heyndrickx, Claire Verschraegen, Nam P. Nguyen, Mark De Ridder, Vincent Vinh-Hung*

*** Correspondence:** Vincent Vinh-Hung: anhxang@gmail.com; vh@onco.be

1 Supplementary Data: original protocol, procedures and investigator comment.

Present page: File page 1.

Part 1: Original protocol. File page 2 to 12.

Part 2: Trial guidance procedure version 02-05-2007b. File page 13 to 25.

Part 3: Trial guidance procedure version 05-Sep-2007. File page 26 to 38.

Part 4: Physiotherapy procedure. File page 39 to 44.

Part 5: Patient's information and consent, French, English, Dutch. File page 45 to 50.

Part 6: Simulation instructions. File page 51 to 52.

Part 7: Investigator commenting clarification. File page 53.

2 Supplementary Figures and Tables: None added.

STUDY PROTOCOL

1. GENERAL INFORMATION

TITLE OF THE RESEARCH PROGRAMME :

Randomized trial comparing conventional vs short-course reduced volume conformal post-surgery radiation treatment in women with stage I or II breast cancer

INSTITUTION(S) OR RESEARCH CENTER where the research will be performed :

Oncologisch Centrum, Universitair Ziekenhuis Brussel, Vrije Universiteit Brussel

NAME OF THE DIRECTOR(S) or of the head of the department(s)* :

Prof. Dr. Guy Storme

PRINCIPAL INVESTIGATOR:

Dr. Vincent Vinh-Hung

RESEARCH DURATION:

Expected 3 Years

2. RESEARCH PROJECT

2.1 Summary of the project:

Tomotherapy is a new radiation therapy system that uses an integrated CT scanner during delivery of radiation treatment to improve the accuracy of the treatment. Furthermore the irradiation is delivered helicoidally allowing highly conformal shaping of dose distribution. However the magnitude of the clinical advantage of using the system in breast cancer is unknown. The purpose of the present study is to investigate whether or not the Tomotherapy can substantially reduce pulmonary and cardiac toxicities, as compared with conventional radiotherapy. This is an important issue that should be investigated to avoid continued exposure of patients to unnecessary toxicities.

2.2 Description of the research project, situation and major steps.

2.1.1 Introduction

- During the last few years evidence has accumulated that post-operative radiotherapy for breast cancer has a favourable impact on survival(*1-8*). The survival impact appears to be sustained with longer follow-up(*9*). However, many issues related to radiation treatment are far from being resolved. A previous overview by the Early Breast Cancer Trialists' Collaborative Group (EBCTCG)(*10*) generated an abundant correspondence that summarizes controversies that are still actual. It was argued that the survival advantage of radiotherapy was related to the inadequacy of surgery(*11*), and contrariwise that the benefit might be rather observed in patients with a small tumor burden (e.g. node-positive after mastectomy with a sufficient number of lymph nodes removed)(*12*). Other points debated were the role of the radiotherapy technique and target(*12,13*), the problem of local recurrence control vs. side effects(*14*), the influence of factors such as tumor size, number of positive nodes and nodes removed, menopausal and hormone receptor status, age, and the histopathology and grade of tumor(*12*).

- We have drawn attention to the fact that the debate highlighted that there is no consensus to explain the survival benefit, and thus no consensus on how the treatment can be improved. Local control has been considered to be the major factor to improve survival, implying that more extensive radiotherapy to breast, chest wall, nodal areas would be required, as well as extensive surgery. But, if toxicity is the major factor, then more parsimonious radiotherapy would be required(*15,16*). In a meta-analysis, Morris et al found that breast-conserving surgery and radiation to the breast conferred a survival advantage compared with mastectomy(*17*). The survival advantage was attributed to the incidental irradiation of the internal mammary chain and lower axilla(*18*). In another meta-analysis, Vinh-Hung and Verschraegen also found a survival advantage with breast conserving

surgery and radiation(*7*). But they attributed the survival advantage to the avoidance of the internal mammary chain and the supraclavicular fossa or the axilla.

- We remarked then that the current evidence was equivocal. It leads to opposed approaches in the daily practice(*19,20*), with consequences on survival that cannot be evaluated until many years. Hence, there is need for additional studies.

- The latest EBCTCG confirmed our findings (*21*). But there has been no progress to elucidate how radiotherapy should be performed. Instead, the EBCTCG equated "four local recurrence avoided, one breast cancer death avoided". Such an equation bears the danger of falling, from one extreme of too little local treatment, to the other extreme of excessive local treatment (*22*). The need for additional studies has not abated.

- We note that new technologies that have advanced the accuracy and the shaping of radiation dose delivery are now available, such as the Novalis, the Tomotherapy, the Topotherapy systems. But despite these technological improvements, breast cancer patients still continue to be treated by radiation techniques with large fields and prolonged fractionation that have remained almost unchanged since the 1980's (*23*). The question that arises is whether or not breast cancer patients should benefit from the new technologies. As had been demonstrated by Van de Steene et al, an impact on survival would require large clinical trials with long follow-up (*9*). However, it has been shown that lung and cardiac toxicities can be detected at 6 months after treatment (*24,25*), or even earlier at 6 weeks post-radiotherapy (*26*), and that these changes predicted long term impairment (*26*). Hence, it is reasonable to consider a single institution study to address the issue of whether or not the new technologies can substantially reduce the pulmonary and the cardiac toxicities of adjuvant radiotherapy.

2.1.2 Objectives

- General objective of the study: To compare accelerated adjuvant radiotherapy with the Tomotherapy system versus conventional post-surgery radiotherapy. The hypothesis tested is that the Tomotherapy treatment will substantially reduce the incidence of pulmonary and cardiac toxicities.
- Primary endpoint: Pulmonary and cardiac toxicities determined by medical imaging and functional tests during follow-up versus pre-treatment evaluation.
- Secondary endpoint(s): Local-regional recurrences as event during follow-up.

2.1.3 Methods

- Study population

  - Disease and disease characteristics
    - Histologically proven invasive breast carcinoma
    - Stage I or II (T1-3N0 or T1-2N1 M0, AJCC/TNM 6th edition)
  - Major inclusion criteria:
    - Informed consent
    - Women
    - $\geq$ 18 years old
    - Surgery with clear margins
    - Pre-operative medical imaging (at least CT, MRI, and/or PET-scan)
    - Remark: type of surgery is not a selection criteria. In our institution, the conventional technique regarding lung/heart is not influenced by the type of surgery.
  - Major exclusion criteria
    - Patients who do not match inclusion criteria
    - Prior breast or thoracic radiotherapy
    - Pregnancy or lactation
    - Fertile patients without effective contraception
    - Psychiatric or addictive disorders
  - Total number of patients in the study: 118
  - Number of Belgian (i.e. treated in Belgium) patients in the study: 118
  - Centers where study will be performed (in a case of a multicenter study, mention specific contribution of each center, including number of patients to be treated in each Belgian center): Oncologisch Centrum, UZ Brussel.
  - Number of patients meeting inclusion/exclusion criteria in your center in the previous year (in case of a multicenter study, describe for each Belgian center): estimated 40.

- General description of the study, including anticipated risks:
  - Prior to surgery: histological confirmation; medical imaging.
  - Localizing markers are placed in case of breast conserving surgery.
  - After surgery, patients are randomized to one of two treatment arms:
  - Arm I: radiotherapy using tangential chest fields, and supraclavicular field in case of nodal involvement, according to our hospital's standard procedure (*27,28*). Dose-fractionation: 50 Gy in 25 fractions over 5 weeks, 2 Gy/fraction. Additional boost 16 Gy in 8 fractions over 2 weeks if breast conserving surgery (verify marker/clip localization) and age <= 70 years.
  - Arm II: radiotherapy using the Tomotherapy system. Target area (breast, thorax wall, nodal areas) delimited according to pre-operative imaging and pathological description. Dose-fractionation (adapted from Whelan et al (*29*)): 42 Gy in 15 fractions over 3 weeks, 2.8 Gy/fraction. Simultaneous boost 0.6 Gy/fraction if breast conserving surgery (computed Equivalent dosis).
  - Physics quality control is integrated during treatment in both arms.
  - Radiotherapy begins within 6 weeks after the last breast surgery. Concurrent or sequential adjuvant systemic treatments are allowed. In case of sequential adjuvant treatment with chemotherapy first, radiotherapy begins within 6 weeks after completion of the adjuvant chemotherapy.

- Quality of life, arm mobility and edema, pulmonary and heart function are assessed prior to radiotherapy, at 1–3 months after completion of radiotherapy, then yearly.
- There is no unexpected anticipated risks with the Tomotherapy. Nevertheless safety monitoring is considered: see Early Stopping below. Note that the possibility of reduced or increased risk of long term secondary tumours is unknown, hence the life-long follow-up (see below).

- Feasibility

  - Anticipated period of inclusion: Three years.
  - Duration of follow-up: Patients are followed at 1 month post-radiotherapy then every 3 months for 3 years, then every 6 months for 3 years, then yearly thereafter (life-long).
  - Statistical hypothesis and how the sample size has been chosen accordingly:
    - Accrual: The estimated incidence of detectable any-grade pulmonary toxicity is 23% (*24*) and cardiac toxicity is 5%–7% (*25*). The hypothesis tested is that the Tomotherapy would reduce the incidence of cumulative toxicity from 25% to 5%. For power 0.80 and significance 0.05, two-sided test, balanced assignment, the accrual required is 118 patients (*30*).
    - Early Stopping: Planned interim analyses yearly. Assessment of unexpected adverse grade 3-4 events, local-regional recurrences, secondary tumours, and any-grade pulmonary-cardiac toxicities (any-grade since these are our primary end points). If the proportion of these pooled events in one arm exceeds the proportion of the pooled events in the other arm with a power of 0.80 and significance of 0.005 (adjustment for multiple comparisons), the study will be stopped after extern consultation (for consideration of power of test, see also (*30*)).
  - Statistical analysis: Comparison of proportions using Fisher's exact test. Time to event using the Kaplan-Meier estimates, comparisons using the log-rank test.

- Expected results and possible implications for clinical oncology
  - It is expected that the Tomotherapy will significantly and substantially reduce any-grade of pulmonary or cardiac toxicities.
  - This would be an important point in considering combined treatment with cytotoxic agents.
  - Confirmation of substantial reduction of toxicities would warrant consideration of a large trial to evaluate the impact on long term survival.

2.3 The 5 most representative publications of the principal investigator that were published in the last ten years.

Verschraegen C, Vinh-Hung V. Effects of radiotherapy and surgery for early breast cancer. Lancet. 2006 May 20;367:1654. *(The correspondence summarizes main issues and recalls the precedence of our researches).*

Woodward WA, Vinh-Hung V, Ueno NT, Cheng YC, Royce M, Tai P, Vlastos G, Wallace AM, Hortobagyi GN, Nieto Y. Prognostic value of nodal ratios in node-positive breast cancer. J Clin Oncol. 2006 Jun 20;24:2910-6. *(Our studies are part of a cooperative network – the INRWG was founded in December 2004 at San Antonio on the initiative of Vincent Vinh-Hung who is currently the secretary).*

Vinh-Hung V, Gordon R. Quantitative target sizes for breast tumor detection prior to metastasis: a prerequisite to rational design of 4D scanners for breast screening. Technol Cancer Res Treat. 2005 Feb;4:11-21. *(Mathematical modelling towards the design of medical imaging equipment).*

Voordeckers M, Van de Steene J, Vinh-Hung V, Storme G. Adjuvant radiotherapy after mastectomy for pT1-pT2 node negative (pN0) breast cancer: is it worth the effort? Radiother Oncol. 2003 Sep;68:227-31. *(Single institution experience in post-mastectomy radiotherapy for stage I breast cancer)*.

Van de Steene J, Vinh-Hung V, Cutuli B, Storme G. Adjuvant radiotherapy for breast cancer: effects of longer follow-up. Radiother Oncol. 2004 Jul;72:35-43. *(Longer follow-up of randomized clinical trials show a survival advantage of radiotherapy).*

3. RESEARCH TEAM

Surnames, first names and titles of the researchers involved in this project.
Expected extent each will contribute to this research (e.g. 70 % of a full-time job):

J. Lamote, G. Verfaillie, surgery, 10%
C. Fontaine, D. Schallier, medical oncology, 10%
J. de Mey, T. Stadnik, C. Breucq, radiology, 20%
H. Everaert, nuclear medicine, 5%
G. Soete, D. Van den Berge, M. Voordeckers, V. Vinh-Hung, radiation oncology, 30%
P. Lievens, rehabilitation medicine, 15%
D. Verellen, K. Tournel, medical physics, 20%

REFERENCES

(1) Ragaz J, Jackson SM, Le N, Plenderleith IH, Spinelli JJ, Basco VE, et al. Adjuvant radiotherapy and chemotherapy in node-positive premenopausal women with breast cancer. N Engl J Med 1997;337:956-62.

(2) Overgaard M, Hansen PS, Overgaard J, Rose C, Andersson M, Bach F, et al. Postoperative radiotherapy in high-risk premenopausal women with breast cancer who receive adjuvant chemotherapy. Danish Breast Cancer Cooperative Group 82b Trial. N Engl J Med 1997;337:949-55.

(3) Overgaard M, Jensen MB, Overgaard J, Hansen PS, Rose C, Andersson M, et al. Postoperative radiotherapy in high-risk postmenopausal breast-cancer patients given adjuvant tamoxifen: Danish Breast Cancer Cooperative Group DBCG 82c randomised trial. Lancet 1999;353:1641-8.

(4) Whelan TJ, Julian J, Wright J, Jadad AR, Levine ML. Does locoregional radiation therapy improve survival in breast cancer? A meta-analysis. J Clin Oncol 2000;18:1220-9.

(5) Van de Steene J, Soete G, Storme G. Adjuvant radiotherapy for breast cancer significantly improves overall survival: the missing link. Radiother Oncol 2000;55:263-72.

(6) Vinh-Hung V, Burzykowski T, Van de Steene J, Storme G, Soete G. Post-surgery radiation in early breast cancer: survival analysis of registry data. Radiother Oncol 2002;64:281-90.

(7) Vinh-Hung V, Verschraegen C, The Breast Conserving Surgery Project. Breast-conserving surgery with or without radiotherapy: pooled-analysis for risks of ipsilateral breast tumor recurrence and mortality. J Natl Cancer Inst 2004;96:115-21. Available at http://jncicancerspectrum.oupjournals.org/cgi/content/full/jnci;96/2/115. [Last accessed: 3-27-2005.]

(8) Vallis KA, Tannock IF. Postoperative radiotherapy for breast cancer: growing evidence for an impact on survival. J Natl Cancer Inst 2004;96:88-9. Available at http://jncicancerspectrum.oupjournals.org/cgi/content/full/jnci;96/2/88. [Last accessed: 1-27-2004.]

(9) Van de Steene J, Vinh-Hung V, Cutuli B, Storme G. Adjuvant radiotherapy for breast cancer: effects of longer follow-up. Radiother Oncol 2004;72:35-43.

(10) Early Breast Cancer Trialists' Collaborative Group (EBCTCG). Favourable and unfavourable effects on long-term survival of radiotherapy for early breast cancer: an overview of the randomised trials. Lancet 2000;355:1757-70.

(11) Atkins CD. Breast cancer survival advantage with radiotherapy. Lancet 2000;356:1269.

(12) Overgaard J, Bartelink H. Breast cancer survival advantage with radiotherapy. Lancet 2000;356:1269-70.

(13) Ragaz J, Spinelli JJ, Coldman AJ. Breast cancer survival advantage with radiotherapy. Lancet 2000;356:1270.

(14) EBCTCG Secretariat. Breast cancer survival advantage with radiotherapy. Lancet 2000;356:1271.

(15) Veronesi U, Gatti G, Luini A, Intra M, Ciocca M, Sanchez D, et al. Full-dose intraoperative radiotherapy with electrons during breast-conserving surgery. Arch Surg 2003;138:1253-6.

(16) Intra M, Orecchia R, Veronesi U. Intraoperative radiotherapy: the debate continues. Lancet Oncol 2004;5:340.

(17) Morris AD, Morris RD, Wilson JF, White J, Steinberg S, Okunieff P, et al. Breast-conserving therapy vs mastectomy in early-stage breast cancer: a meta-analysis of 10-year survival. Cancer J Sci Am 1997;3:6-12.

(18) Haffty BG, Ward BA. Is breast-conserving surgery with radiation superior to mastectomy in selected patients? Cancer J Sci Am 1997;3:2-3.

(19) Taghian A, Jagsi R, Makris A, Goldberg S, Ceilley E, Grignon L, et al. Results of a survey regarding irradiation of internal mammary chain in patients with breast cancer: Practice is culture driven rather than evidence based. Int J Radiat Oncol Biol Phys 2004;60:706-14.

(20) White J, Moughan J, Pierce LJ, McNeese M, Owen J, Wilson JF. Comparison of practice patterns and treatment techniques for regional nodal irradiation with breast conservation vs. postmastectomy in stage I-II breast cancer. Abstract 2071. Int J Radiat Oncol Biol Phys 2004;60, Suppl 1:S378.

(21) Early Breast Cancer Trialists' Collaborative Group (EBCTCG). Effects of radiotherapy and of differences in the extent of surgery for early breast cancer on local recurrence and 15-year survival: an overview of the randomised trials. Lancet 2005;366:2087-106.

(22) Verschraegen C, Vinh-Hung V. Effects of radiotherapy and surgery for early breast cancer. Lancet 2006;367:1654-5.

(23) Gebski V, Lagleva M, Keech A, Simes J, Langlands AO. Survival effects of postmastectomy adjuvant radiation therapy using biologically equivalent doses: a clinical perspective. J Natl Cancer Inst 2006;98:26-38.

(24) Wennberg B, Gagliardi G, Sundbom L, Svane G, Lind P. Early response of lung in breast cancer irradiation: radiologic density changes measured by CT and symptomatic radiation pneumonitis. Int J Radiat Oncol Biol Phys 2002;52:1196-206.

(25) Lind PA, Pagnanelli R, Marks LB, Borges-Neto S, Hu C, Zhou SM, et al. Myocardial perfusion changes in patients irradiated for left-sided breast cancer and correlation with coronary artery distribution. Int J Radiat Oncol Biol Phys 2003;55:914-20.

(26) Tokatli F, Kaya M, Kocak Z, Ture M, Mert S, Unlu E, et al. Sequential pulmonary effects of radiotherapy detected by functional and radiological end points in women with breast cancer. Clin Oncol (R Coll Radiol) 2005;17:39-46.

(27) Voordeckers M, Van de Steene J, Vinh-Hung V, Storme G. Adjuvant radiotherapy after mastectomy for pT1-pT2 node negative (pN0) breast cancer: is it worth the effort? Radiother Oncol 2003;68:227-31.

(28) Voordeckers M, Vinh-Hung V, Van de Steene J, Lamote J, Storme G. The lymph node ratio as prognostic factor in node-positive breast cancer. Radiother Oncol 2004;70:225-30.

(29) Whelan T, MacKenzie R, Julian J, Levine M, Shelley W, Grimard L, et al. Randomized trial of breast irradiation schedules after lumpectomy for women with lymph node-negative breast cancer. J Natl Cancer Inst 2002;94:1143-50.

(30) Vinh-Hung V. Power of test comparing independent proportions. Comput Biol Med 1986;16:39-43.

Randomized trial of Tomotherapy in breast cancer

Patient	

Inclusion criteria	(all must be *yes* for eligibility)
Histologically proven breast carcinoma	yes / no
Stage I or II (T1-3N0 or T1-2N1 M0)	yes / no
Surgery with clear margins	yes / no
Pre-operative imaging (at least CT, MRI, and/or PET)	yes / no
Woman	yes / no
Age >= 18	yes / no
Signed informed consent	yes / no

Exclusion criteria	(all must be *no* for eligibility)
Prior breast or thoracic radiotherapy	yes / no
Pregnancy or lactation	yes / no
Fertile patient without effective contraception	yes / no
Psychiatric or addictive disorder	yes / no

Randomisation and Eligibility Check	Allocation
Please call Sven D'Haese or Marleen Van Roy, Tel. 6145	Classic / Tomo

Prior to radiotherapy	Date
Echocardiography	
Lung function tests.<br>Co-investigator: Dr.<br>Please call 6010 for appointment.<br>Appointment request form should specify:<br>• Spirometry<br>• Lung volumes measurements<br>• Lung diffusion	
QLQ C-30	
QLQ BR-23	

At simulation		
Clinical Measurements	Right	Left
- Shoulder mobility (arm abduction)		
- Circumference mid-arm		
- Circumference proximal forearm		
- Circumference wrist		
Pictures		
- Front, hands on the hips		
- Front, hands on the head		
- Profile, hands on the head		

Simulation

Patients randomized to conventional RT:
- Classic simulation/planning/treatment.

Patients randomized to tomotherapy:
- **Mastectomy** patients: **supine**, arms above thorax.
- **Tumorectomy** patients: double simulation **supine and prone**, arms above thorax, except:
    1. If problem of time constraint/appointments allowing only a single simulation-planning: prone is preferred.
    2. If prone uncomfortable for patient: supine only.

CT acquisition

CT in treatment position.
Double CT in prone **and** in supine position only for *Tumorectomy patients randomized to Tomotherapy* (see Simulation above).
The CT-scan should start at or above the mandible and extend to at least 5 cm below the inframammary fold. The entire lungs should be included. A scan thickness of 3 mm should be employed. The CT-scans will be acquired without contrast.

Contours

**CTV**	
CTV1 (=breast and/or chest wall)	*After breast conserving surgery*, the CTV1 (=breast and chest wall) will be defined by the conserved breast, limited for lateral and cranio-caudal boundaries by clinical assessment, anteriorly 5 mm below the skin surface except in case of dermis involvement on initial presentation or on pathological report, and posteriorly to the fascia muscularis except in case of muscle involvement.<br>*After mastectomy*, the CTV1 (=chest wall) will be defined for lateral and cranio-caudal boundaries by clinical assessment, based on preoperative data and contralateral breast anatomy, anteriorly 5 mm below the skin except in case of dermis involvement on initial presentation or pathological report, and posteriorly to the fascia muscularis except in case of muscle involvement.<br>In case of dermis or muscle involvement, the skin or muscle will be included in the CTV1 but without extending beyond the air-tissue or lung-tissue interface.
CTVb (=boost)	The CTVb (boost) will be defined by uniformly expanding the excision cavity volume (tumor bed) by 7 mm. Pre-operative imaging, surgical-pathological report, and surgical clips if available should be used to help define the boundaries of the CTVb. The CTVb will not extend beyond 5 mm below the skin surface or beyond the fascia muscularis except in case of skin/muscle involvement.
CTVn (=nodal areas)	In case of axillar-supraclavicular irradiation, the CTVn (= nodal areas) will be defined by countouring the axilla, supra and infraclavicular fossa.<br><br>The axilla including levels I, II and III are defined laterally, posteriorly and medially to the pectoralis minor muscle respectively.<br><br>Supraclavicular fossa is limited anteriorly by the deep surface of the sternocleidomastoid muscle and the deep cervical fascia, posteriorly and inferiorly by the subclavian artery, above, posteriorly and medially by the carotid artery and the internal jugular vein, posteriorly and laterally by the anterior and medial border of the anterior scalene muscle, medially by the lateral edge of the trachea excluding the thyroid gland and cartilage, and laerally by the clavicle.<br><br>Infraclavicular fossa is limited above by the superior limit of the minor pectoralis muscle, and below by the insertion of the clavicle into the manubrium, laterally by the medial border of the minor pectoralis muscle, medially by the lateral edge of the clavicle, anteriorly by the deep surface of the major pectoralis muscle and posteriorly by the subclavian-axillary artery.<br><br>In all cases the CTVn will be limited to 5 mm from the skin surface except in cases of dermis/skin involvement.
CTVhrn (=high risk nodes)	The CTVhrn (high risk nodes) correspond to pre-operative nodal GTV's, defined as macroscopically enlarged lymph nodes. These should be unequivocally identified as clinical-radiological involved lymph nodes with pathological confirmation.

**PTV**	
PTV1	An expansion of 5 mm around the CTV1 is required with the constraints:<br>- Not beyond 4 mm below the skin surface, except in case of documented dermis involvement whereas an expansion of <=5 mm beyond the skin might be allowed.<br>- Not beyond the lung-tissue interface, except in case of documented muscle involvement whereas an expansion of <= 5mm into the lung might be allowed.
PTVb	An expansion of 5 mm around the CTVb is required with the same constraints/exceptions as PTV1.
PTVn	An expansion of 5 mm around the CTVn is required with the same constraints/exceptions as PTV1.
PTVhrn	An expansion of 5 mm around the CTVhrn is required with the same constraints/exceptions as PTV1.

**OAR**	
Heart	For the heart delineation, the cranial extent of the heart includes the infundibulum of the right ventricule, the right atrium, and the right atrium auricle but excludes the pulmonary trunk, the ascending aorta and the superior vena cava. The lowest external contour of the heart is the caudal border of the myocardium. The pericardium is excluded from the heart.
Lungs	The lungs are automatically outlined and checked manually on each slice.
Thyroid	Manual anatomic delineation.
Contralateral breast	Manual anatomic delineation.
Spinal cord expanded	Manual anatomic delineation, then expanded 5 mm in all directions.
Esophagus	Manual anatomic delineation.

Dose prescription

The doses/planning/technique prescribed to patients allocated to *classic radiotherapy* are as described in Voordeckers et al, 2003, and Voordeckers et al, 2004.

[Voordeckers M, Van de Steene J, Vinh-Hung V, Storme G. Adjuvant radiotherapy after mastectomy for pT1-pT2 node negative (pN0) breast cancer: is it worth the effort? Radiother Oncol. 2003 Sep;68(3):227-31].

[Voordeckers M, Vinh-Hung V, Van de Steene J, Lamote J, Storme G. The lymph node ratio as prognostic factor in node-positive breast cancer. Radiother Oncol. 2004 Mar;70(3):225-30].

UZ Brussel protocols MA…

The doses prescribed to patients allocated to *Tomotherapy* are:

- **Mastectomy** patients: **42 Gy** to **PTV1**, **PTVn**, **PTVhrn**, in 15 fractions, 2.8 Gy/fraction, 5 fractions/week. The dose will be prescribed on the 100% isodose.
- **Tumorectomy** patients: **42 Gy** to **PTV1**, **PTVn**, **PTVhrn**, in 15 fractions, 2.8 Gy/fraction, 5 fractions/week. The dose will be prescribed on the 100% isodose. Concomitant boost to **51 Gy** (3.4 Gy/fraction) to **PTVb**.

UZ Brussel protocol …

Planning

Record DVH for:

heart, ipsilateral lung, contralateral breast, CTV1, CTVb, CTVhrn, PTVn.

Record for each structure:

Min, max, average, median, upper and lower quartile of doses delivered.

	Heart	Cont lung	Ipsi lung	CTVb	CTV hrn	Spinal cord	Esophagus	Thyroid	Cont Breast	PTVb	PTV hrn	CTV1	PTV1	CTVn	PTVn
Min (Gy)															
Max (Gy)															
Avg (Gy)															
Med (Gy)															
LowQ (Gy)															
UppQ (Gy)															

Record also Absolute volumes and Effective constraint values delivered (cf the following table Dose Constraints and Priorities).

Dose constraints and priorities

Priority	Structure	Record absolute volume of structure	Constraint/Tolerance	Record effective values delivered
1	Heart		V2Gy <20%<br>V5Gy <10%<br>V8Gy < 5%<br>V17Gy < 2%	
2	Contralateral lung		V2Gy < 10%<br>V5Gy < 5%	
3	Ipsilateral lung		V5Gy < 15%-20%<br>V8Gy < 10%-15%<br>V10Gy < 8%-10%<br>V17Gy < 5%-7%<br>V25Gy < 2%-5%	
4	CTVb		97%-103% of prescribed *boost* dose (49.5 Gy - 52.5 Gy)	
5	CTVhrn		97%-103% of prescribed dose (40.7 Gy - 43.3 Gy)	
6	Spinal cord		V10Gy < 20%<br>Max 20 Gy	
7	Esophagus		V10Gy < 30%<br>V20Gy < 20%<br>Max 30 Gy	
8	Thyroid		V20Gy < 30%<br>Max 35Gy	
9	Contralateral breast		V5Gy < 25%<br>V10Gy < 5%	
10	PTVb		95%-105% of prescribed *boost* dose (48.5 Gy – 53.5 Gy)	
11	PTVhrn		95%-105% of prescribed dose (40 Gy – 44 Gy)	
12	CTV1		95%-105% of prescribed dose (40 Gy – 44 Gy)	
13	PTV1		95%-105% of prescribed dose (40 Gy – 44 Gy)	
14	CTVn		95%-105% of prescribed dose (40 Gy – 44 Gy)	
15	PTVn		95%-105% of prescribed dose (40 Gy – 44 Gy)	

Last day of Radiotherapy

Clinical events/symptoms that occurred during the radiotherapy

If no event/symptom, record date of examination and mention "No".

Date	Event during radiotherapy

End of radiotherapy

Skin toxicity grade
Other events/symptoms, list below:

Follow-up at 1-3 months post-radiotherapy

Planned examinations

	Date
Echocardiography	
Lung function tests (specifications cf page 2)	
QLQ C-30	
QLQ BR-23	

Clinical Measurements	Right	Left
- Shoulder mobility (arm abduction)		
- Circumference mid-arm		
- Circumference proximal forearm		
- Circumference wrist		
Pictures		
- Front, hands on the hips		
- Front, hands on the head		
- Profile, hands on the head		

Adverse events

Medical condition (for example pneumonia, infarct, etc)	Date occurrence	Diagnosis based on	Evolution (if cured: date of cure)

Recurrences

Site of recurrence	Date recurrence	Diagnosis based on	Size of recurrence (if measurable)

Follow-up at year 1 post-radiotherapy

Planned examinations

	Date
Echocardiography	
Lung function tests (specifications cf page 2)	
QLQ C-30	
QLQ BR-23	

Clinical Measurements	Right	Left
- Shoulder mobility (arm abduction)		
- Circumference mid-arm		
- Circumference proximal forearm		
- Circumference wrist		
Pictures		
- Front, hands on the hips		
- Front, hands on the head		
- Profile, hands on the head		

Adverse events

Medical condition (for example pneumonia, infarct, etc)	Date occurrence	Diagnosis based on	Evolution (if cured: date of cure)

Recurrences

Site of recurrence	Date recurrence	Diagnosis based on	Size of recurrence (if measurable)

Follow-up at year 2 post-radiotherapy

Planned examinations

	Date
Echocardiography	
Lung function tests (specifications cf page 2)	
QLQ C-30	
QLQ BR-23	

Clinical Measurements	Right	Left
- Shoulder mobility (arm abduction)		
- Circumference mid-arm		
- Circumference proximal forearm		
- Circumference wrist		
Pictures		
- Front, hands on the hips		
- Front, hands on the head		
- Profile, hands on the head		

Adverse events

Medical condition (for example pneumonia, infarct, etc)	Date occurrence	Diagnosis based on	Evolution (if cured: date of cure)

Recurrences

Site of recurrence	Date recurrence	Diagnosis based on	Size of recurrence (if measurable)

Follow-up at year 3 post-radiotherapy

Planned examinations

	Date
Echocardiography	
Lung function tests (specifications cf page 2)	
QLQ C-30	
QLQ BR-23	

Clinical Measurements	Right	Left
- Shoulder mobility (arm abduction)		
- Circumference mid-arm		
- Circumference proximal forearm		
- Circumference wrist		
Pictures		
- Front, hands on the hips		
- Front, hands on the head		
- Profile, hands on the head		

Adverse events

Medical condition (for example pneumonia, infarct, etc)	Date occurrence	Diagnosis based on	Evolution (if cured: date of cure)

Recurrences

Site of recurrence	Date recurrence	Diagnosis based on	Size of recurrence (if measurable)

Follow-up at year … post-radiotherapy

Examinations (per protocol not required after 3 years)

	Date
Echocardiography	
Lung function tests (specifications cf page 2)	
QLQ C-30	
QLQ BR-23	

Clinical Measurements	Right	Left
- Shoulder mobility (arm abduction)		
- Circumference mid-arm		
- Circumference proximal forearm		
- Circumference wrist		
Pictures		
- Front, hands on the hips		
- Front, hands on the head		
- Profile, hands on the head		

Adverse events

Medical condition (for example pneumonia, infarct, etc)	Date occurrence	Diagnosis based on	Evolution (if cured: date of cure)

Recurrences

Site of recurrence	Date recurrence	Diagnosis based on	Size of recurrence (if measurable)

Randomized trial of Tomotherapy in breast cancer

Patient	

Inclusion criteria	
Histologically proven breast carcinoma	yes / no
Stage I or II (T1-3N0 or T1-2N1 M0)	yes / no
Surgery with clear margins	yes / no
Pre-operative imaging (at least CT, MRI, and/or PET)*<br>*mandatory if pN1 and/or tumorectomy without clips	yes / no
Woman	yes / no
Age >= 18	yes / no
Signed informed consent	yes / no

Exclusion criteria	
Prior breast or thoracic radiotherapy	yes / no
Pregnancy or lactation	yes / no
Fertile patient without effective contraception	yes / no
Psychiatric or addictive disorder	yes / no

Randomisation and Eligibility Check	Allocation
Please call Sven D'Haese, Tel. 6145<br>or Marleen Van Roy, Tel. 6146	Classic / Tomo

Prior to radiotherapy	Date
Echocardiography.<br>Co-investigator: Dr Caroline Weytjens.<br>Please call 6010 for appointment.<br>Exam location: Polikliniek Cardiologie.	
Lung function tests.<br>Co-investigator: Daniel Schuermann.<br>Please call 6346 for appointment.<br>Appointment request form should specify:<br>• Spirometry<br>• Lung volumes measurements<br>• Lung diffusion<br>Exam location: Polikliniek Pneumologie.	
Functional-kinesitherapeutic evaluation.<br>Co-investigator: Eva Swinnen.<br>Please call 4530 for appointment.<br>Exam location: Borstkliniek	
QLQ C-30 and BR-23.<br>Give forms to patient.	

At simulation		
Clinical Measurements	Right	Left
- Shoulder mobility (arm abduction)		
- Circumference mid-arm		
- Circumference proximal forearm		
- Circumference wrist		
Pictures		
- Front, hands on the hips		
- Front, hands on the head		
- Profile, hands on the head		

Simulation

Patients randomized to conventional RT:
- Classic simulation/planning/treatment.

Patients randomized to tomotherapy:
- **Mastectomy** patients: **supine**, arms above thorax.
- **Tumorectomy** patients: double simulation **supine and prone**, arms above thorax, except:
    1. If problem of time constraint/appointments allowing only a single simulation-planning: prone is preferred.
    2. If prone uncomfortable for patient: supine only.

CT acquisition

CT in treatment position.
Double CT in prone **and** in supine position only for *Tumorectomy patients randomized to Tomotherapy* (see Simulation above).
The CT-scan should start at or above the mandible and extend to at least 5 cm below the inframammary fold. The entire lungs should be included. A scan thickness of 3 mm should be employed. The CT-scans will be acquired without contrast.

Contours

**CTV**	
CTV1 (=breast and/or chest wall)	*After breast conserving surgery*, the CTV1 (=breast and chest wall) will be defined by the conserved breast, limited for lateral and cranio-caudal boundaries by clinical assessment, anteriorly 5 mm below the skin surface except in case of dermis involvement on initial presentation or on pathological report, and posteriorly to the fascia muscularis except in case of muscle involvement.<br>*After mastectomy*, the CTV1 (=chest wall) will be defined for lateral and cranio-caudal boundaries by clinical assessment, based on preoperative data and contralateral breast anatomy, anteriorly 5 mm below the skin except in case of dermis involvement on initial presentation or pathological report, and posteriorly to the fascia muscularis except in case of muscle involvement.<br>In case of dermis or muscle involvement, the skin or muscle will be included in the CTV1 but without extending beyond the air-tissue or lung-tissue interface.
CTVb (=boost)	The CTVb (boost) will be defined by uniformly expanding the excision cavity volume (tumor bed) by 7 mm. Pre-operative imaging, surgical-pathological report, and surgical clips if available should be used to help define the boundaries of the CTVb. The CTVb will not extend beyond 5 mm below the skin surface or beyond the fascia muscularis except in case of skin/muscle involvement.
CTVn (=nodal areas)	In case of axillar-supraclavicular irradiation, the CTVn (= nodal areas) will be defined by countouring the axilla, supra and infraclavicular fossa.<br><br>The axilla including levels I, II and III are defined laterally, posteriorly and medially to the pectoralis minor muscle respectively.<br><br>Supraclavicular fossa is limited anteriorly by the deep surface of the sternocleidomastoid muscle and the deep cervical fascia, posteriorly and inferiorly by the subclavian artery, above, posteriorly and medially by the carotid artery and the internal jugular vein, posteriorly and laterally by the anterior and medial border of the anterior scalene muscle, medially by the lateral edge of the trachea excluding the thyroid gland and cartilage, and laerally by the clavicle.<br><br>Infraclavicular fossa is limited above by the superior limit of the minor pectoralis muscle, and below by the insertion of the clavicle into the manubrium, laterally by the medial border of the minor pectoralis muscle, medially by the lateral edge of the clavicle, anteriorly by the deep surface of the major pectoralis muscle and posteriorly by the subclavian-axillary artery.<br><br>In all cases the CTVn will be limited to 5 mm from the skin surface except in cases of dermis/skin involvement.
CTVhrn (=high risk nodes)	The CTVhrn (high risk nodes) correspond to pre-operative nodal GTV's, defined as macroscopically enlarged lymph nodes. These should be unequivocally identified as clinical-radiological involved lymph nodes with pathological confirmation.

**PTV**	
PTV1	An expansion of 5 mm around the CTV1 is required with the constraints:<br>- Not beyond 4 mm below the skin surface, except in case of documented dermis involvement whereas an expansion of <=5 mm beyond the skin might be allowed.<br>- Not beyond the lung-tissue interface, except in case of documented muscle involvement whereas an expansion of <= 5mm into the lung might be allowed.
PTVb	An expansion of 5 mm around the CTVb is required with the same constraints/exceptions as PTV1.
PTVn	An expansion of 5 mm around the CTVn is required with the same constraints/exceptions as PTV1.
PTVhrn	An expansion of 5 mm around the CTVhrn is required with the same constraints/exceptions as PTV1.

**OAR**	
Heart	For the heart delineation, the cranial extent of the heart includes the infundibulum of the right ventricule, the right atrium, and the right atrium auricle but excludes the pulmonary trunk, the ascending aorta and the superior vena cava. The lowest external contour of the heart is the caudal border of the myocardium. The pericardium is excluded from the heart.
Lungs	The lungs are automatically outlined and checked manually on each slice.
Thyroid	Manual anatomic delineation.
Contralateral breast	Manual anatomic delineation.
Spinal cord expanded	Manual anatomic delineation, then expanded 5 mm in all directions.
Esophagus	Manual anatomic delineation.

Dose prescription

The doses/planning/technique prescribed to patients allocated to *classic radiotherapy* are as described in Voordeckers et al, 2003, and Voordeckers et al, 2004.

[Voordeckers M, Van de Steene J, Vinh-Hung V, Storme G. Adjuvant radiotherapy after mastectomy for pT1-pT2 node negative (pN0) breast cancer: is it worth the effort? Radiother Oncol. 2003 Sep;68(3):227-31].

[Voordeckers M, Vinh-Hung V, Van de Steene J, Lamote J, Storme G. The lymph node ratio as prognostic factor in node-positive breast cancer. Radiother Oncol. 2004 Mar;70(3):225-30].

UZ Brussel protocols MA…

The doses prescribed to patients allocated to *Tomotherapy* are:

- **Mastectomy** patients: **42 Gy** to **PTV1**, **PTVn**, **PTVhrn**, in 15 fractions, 2.8 Gy/fraction, 5 fractions/week. The dose will be prescribed on the 100% isodose.
- **Tumorectomy** patients: **42 Gy** to **PTV1**, **PTVn**, **PTVhrn**, in 15 fractions, 2.8 Gy/fraction, 5 fractions/week. The dose will be prescribed on the 100% isodose. Concomitant boost to **51 Gy** (3.4 Gy/fraction) to **PTVb**.

UZ Brussel protocol …

Planning

Record DVH for:

heart, ipsilateral lung, contralateral breast, CTV1, CTVb, CTVhrn, PTVn.

Record for each structure:

Min, max, average, median, upper and lower quartile of doses delivered.

	Heart	Cont lung	Ipsi lung	CTVb	CTV hrn	Spinal cord	Esoph agus	Thyroi d	Cont Breast	PTVb	PTV hrn	CTV1	PTV1	CTVn	PTVn
Min (Gy)															
Max (Gy)															
Avg (Gy)															
Med (Gy)															
LowQ (Gy)															
UppQ (Gy)															

Record also Absolute volumes and Effective constraint values delivered (cf the following table Dose Constraints and Priorities).

Dose constraints and priorities

Priority	Structure	Record absolute volume of structure	Constraint/Tolerance	Record effective values delivered
1	Heart		V2Gy <20%<br>V5Gy <10%<br>V8Gy < 5%<br>V17Gy < 2%	
2	Contralateral lung		V2Gy < 10%<br>V5Gy < 5%	
3	Ipsilateral lung		V5Gy < 15%-20%<br>V8Gy < 10%-15%<br>V10Gy < 8%-10%<br>V17Gy < 5%-7%<br>V25Gy < 2%-5%	
4	CTVb		97%-103% of prescribed *boost* dose (49.5 Gy - 52.5 Gy)	
5	CTVhrn		97%-103% of prescribed dose (40.7 Gy - 43.3 Gy)	
6	Spinal cord		V10Gy < 20%<br>Max 20 Gy	
7	Esophagus		V10Gy < 30%<br>V20Gy < 20%<br>Max 30 Gy	
8	Thyroid		V20Gy < 30%<br>Max 35Gy	
9	Contralateral breast		V5Gy < 25%<br>V10Gy < 5%	
10	PTVb		95%-105% of prescribed *boost* dose (48.5 Gy – 53.5 Gy)	
11	PTVhrn		95%-105% of prescribed dose (40 Gy – 44 Gy)	
12	CTV1		95%-105% of prescribed dose (40 Gy – 44 Gy)	
13	PTV1		95%-105% of prescribed dose (40 Gy – 44 Gy)	
14	CTVn		95%-105% of prescribed dose (40 Gy – 44 Gy)	
15	PTVn		95%-105% of prescribed dose (40 Gy – 44 Gy)	

Last day of Radiotherapy

Clinical events/symptoms that occurred during the radiotherapy

If no event/symptom, record date of examination and mention "No".

Date	Event during radiotherapy

End of radiotherapy

Skin toxicity grade
Other events/symptoms, list below:

Follow-up at 1-3 months post-radiotherapy

Planned examinations

	Date
Echocardiography	
Lung function tests (specifications cf page 2)	
QLQ C-30	
QLQ BR-23	

Clinical Measurements	Right	Left
- Shoulder mobility (arm abduction)		
- Circumference mid-arm		
- Circumference proximal forearm		
- Circumference wrist		
Pictures		
- Front, hands on the hips		
- Front, hands on the head		
- Profile, hands on the head		

Adverse events

Medical condition (for example pneumonia, infarct, etc)	Date occurrence	Diagnosis based on	Evolution (if cured: date of cure)

Recurrences

Site of recurrence	Date recurrence	Diagnosis based on	Size of recurrence (if measurable)

Follow-up at year 1 post-radiotherapy

Planned examinations

	Date
Echocardiography	
Lung function tests (specifications cf page 2)	
QLQ C-30	
QLQ BR-23	

Clinical Measurements	Right	Left
- Shoulder mobility (arm abduction)		
- Circumference mid-arm		
- Circumference proximal forearm		
- Circumference wrist		
Pictures		
- Front, hands on the hips		
- Front, hands on the head		
- Profile, hands on the head		

Adverse events

Medical condition (for example pneumonia, infarct, etc)	Date occurrence	Diagnosis based on	Evolution (if cured: date of cure)

Recurrences

Site of recurrence	Date recurrence	Diagnosis based on	Size of recurrence (if measurable)

Follow-up at year 2 post-radiotherapy

Planned examinations

	Date
Echocardiography	
Lung function tests (specifications cf page 2)	
QLQ C-30	
QLQ BR-23	

Clinical Measurements	Right	Left
- Shoulder mobility (arm abduction)		
- Circumference mid-arm		
- Circumference proximal forearm		
- Circumference wrist		
Pictures		
- Front, hands on the hips		
- Front, hands on the head		
- Profile, hands on the head		

Adverse events

Medical condition (for example pneumonia, infarct, etc)	Date occurrence	Diagnosis based on	Evolution (if cured: date of cure)

Recurrences

Site of recurrence	Date recurrence	Diagnosis based on	Size of recurrence (if measurable)

Follow-up at year 3 post-radiotherapy

Planned examinations

	Date
Echocardiography	
Lung function tests (specifications cf page 2)	
QLQ C-30	
QLQ BR-23	

Clinical Measurements	Right	Left
- Shoulder mobility (arm abduction)		
- Circumference mid-arm		
- Circumference proximal forearm		
- Circumference wrist		
Pictures		
- Front, hands on the hips		
- Front, hands on the head		
- Profile, hands on the head		

Adverse events

Medical condition (for example pneumonia, infarct, etc)	Date occurrence	Diagnosis based on	Evolution (if cured: date of cure)

Recurrences

Site of recurrence	Date recurrence	Diagnosis based on	Size of recurrence (if measurable)

Follow-up at year … post-radiotherapy

Examinations (per protocol not required after 3 years)

	Date
Echocardiography	
Lung function tests (specifications cf page 2)	
QLQ C-30	
QLQ BR-23	

Clinical Measurements	Right	Left
- Shoulder mobility (arm abduction)		
- Circumference mid-arm		
- Circumference proximal forearm		
- Circumference wrist		
Pictures		
- Front, hands on the hips		
- Front, hands on the head		
- Profile, hands on the head		

Adverse events

Medical condition (for example pneumonia, infarct, etc)	Date occurrence	Diagnosis based on	Evolution (if cured: date of cure)

Recurrences

Site of recurrence	Date recurrence	Diagnosis based on	Size of recurrence (if measurable)

Protocol Physiotherapeutic Evaluation:

Patient	
File number	
Operation-Radiation side	

Evaluation	*Data*
1) Follow-up pre-radiotherapy	
2) Follow-up 1-3 months post-radiotherapy	
3) Follow-up 1 year post-radiotherapy	
4) Follow-up 2 years post-radiotherapy	
5) Follow-up 3 years post-radiotherapy	

Measurements	***Operation-Radiation side (ORS)***	***Non-Operation-Radiation side (NORS)***	***Difference ORS-NORS***
1) Functional mobility			
Abduction<br>(maximum passive arm abduction, extension elbow, in degrees)			
Retroflexion<br>(maximum passive retroflexion shoulder, extension elbow, in degrees)			
Anteflexion<br>(maximum passive anteflexion shoulder, extension elbow, in degrees)			
Scapulae distance<br>(distance between angulus inferior scapula and spine at same high with arm in 90° anteflexion, in centimetres)			
Scapula alatae<br>(tilt angelus inferior scapulae, arms from 0° to 90° anteflexion, Yes or No)			
Endorotation<br>(number vertebrae between C7 and thumb)			

2) Pictures (X? meter distance)			
Front, sit, arms on table, extension elbow			
Front, arms 0° anteflexion			
Front, hands on head			
Profile, hands on head			
Back, arms 0° anteflexion			
Back, arms 90° anteflexion			
3) Size edema (circumference)			
1 (15cm above elbow)			
2 (10cm above elbow)			
3 (5cm below olecranon)			
4 (10 cm below olecranon)			
5 (15 cm below olecranon)			
4) Hardness edema (tonometry)			
1 (ventral, 10cm above elbow)			
2 (ventral, 5cm above elbow))			
3 (ventral, 10cm below elbow)			
4 (dorsal, 10cm above olecranon)			
5 (dorsal, 5cm below olecranon)			
6 (dorsal, 10cm below olecranon)			
7 (dorsal, hand)			

5) Edema (Echography)			
Thickness dermis			
1 (ventral, 10cm above elbow)			
2 (ventral, 5cm above elbow)			
3 (ventral, 10cm below elbow)			
4 (dorsal, 10cm above olecranon)			
5 (dorsal, 5cm below olecranon)			
6 (dorsal, 10cm below olecranon)			
7 (dorsal, hand)			
Thickness subcutis			
1 (ventral, 10cm above elbow)			
2 (ventral, 5cm above elbow))			
3 (ventral, 10cm below elbow)			
4 (dorsal, 10cm above olecranon)			
5 (dorsal, 5cm below olecranon)			
6 (dorsal, 10cm below olecranon)			
7 (dorsal, hand)			
Echogenicity dermis			
1 (ventral, 10cm above elbow)			
2 (ventral, 5cm above elbow))			
3 (ventral, 10cm below elbow)			
4 (dorsal, 10cm above olecranon)			
5 (dorsal, 5cm below olecranon)			
6 (dorsal, 10cm below olecranon)			
7 (dorsal, hand)			
Echogenicity subcutis			
1 (ventral, 10cm above elbow)			
2 (ventral, 5cm above elbow))			
3 (ventral, 10cm below elbow)			
4 (dorsal, 10cm above olecranon)			
5 (dorsal, 5cm below olecranon)			
6 (dorsal, 10cm below olecranon)			
7 (dorsal, hand)			

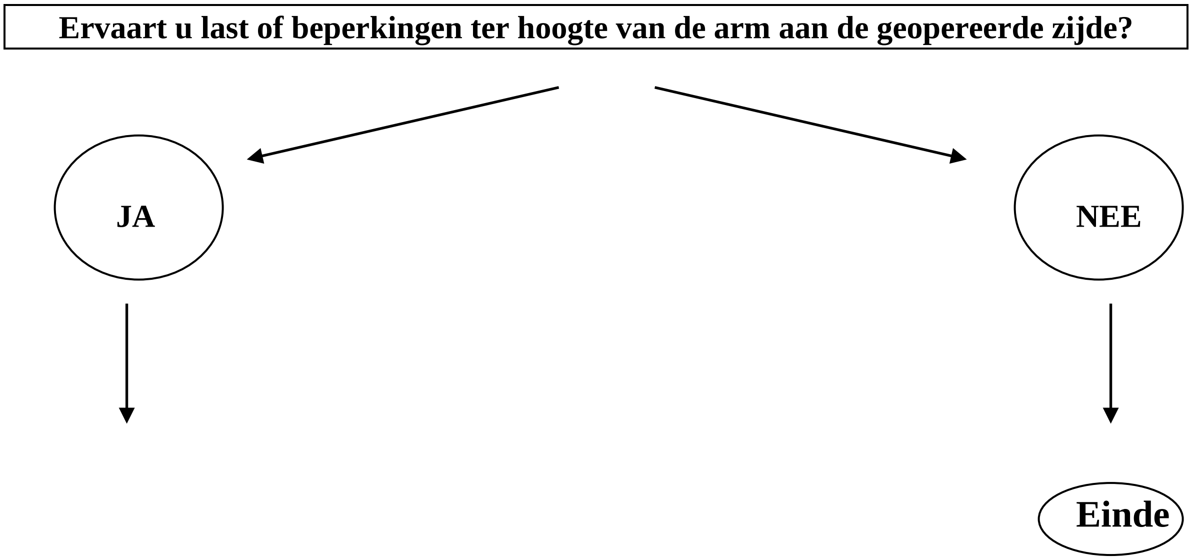

Vragenlijst 2:

Tintelingen:

	- (afwezig)	+ (weinig)	++ (tamelijk)	+++ (uitermate)
**t.h.v. bovenarm**				
**t.h.v. onderarm**				
**t.h.v. hand**				
**t.h.v. borst**				
**t.h.v. flank**				
**t.h.v. schouderblad**				

Gevoelloosheid:

	- (afwezig)	+ (weinig)	++ (tamelijk)	+++ (uitermate)
**t.h.v. bovenarm**				
**t.h.v. onderarm**				
**t.h.v. hand**				
**t.h.v. borst**				
**t.h.v. flank**				
**t.h.v. schouderblad**				

Zwaar gevoel:

	- (afwezig)	+ (weinig)	++ (tamelijk)	+++ (uitermate)
**t.h.v. bovenarm**				
**t.h.v. onderarm**				
**t.h.v. hand**				
**t.h.v. borst**				
**t.h.v. flank**				
**t.h.v. schouderblad**				

Warmte:

	- (afwezig)	+ (weinig)	++ (tamelijk)	+++ (uitermate)
**t.h.v. bovenarm**				
**t.h.v. onderarm**				
**t.h.v. hand**				
**t.h.v. borst**				
**t.h.v. flank**				
**t.h.v. schouderblad**				

Pijn:

	- (afwezig)	+ (weinig)	++ (tamelijk)	+++ (uitermate)
**t.h.v. bovenarm**				
**t.h.v. onderarm**				
**t.h.v. hand**				
**t.h.v. borst**				
**t.h.v. flank**				
**t.h.v. schouderblad**				

Andere gewaarwordingen:

	-(afwezig)	+ (weinig)	++ (tamelijk)	+++ (uitermate)
**t.h.v. bovenarm**				
**t.h.v. onderarm**				
**t.h.v. hand**				
**t.h.v. borst**				
**t.h.v. flank**				
**t.h.v. schouderblad**				

In welke mate heeft u beperkingen bij het uitvoeren van onderstaande taken?

	-(geen beperkingen)	+ (uitvoerbaar, met lichte aanpassingen)	++ (in beperkte mate nog uitvoerbaar)	+++ (niet in staat uit te voeren)
**Huishouden**				
**Werk**				
**Hobby's**				
**Andere activiteiten**				

Physiotherapist = “Blind”

Materiaal:	-Lintmeter	*OK of aankopen: Gymna.
	-Tonometer (2?):	*Aankopen: Tonometer Vodder School
		*(2de model zoeken)
	-Goniometer:	*Aankopen: Gymna: Goniometer grote gewrichten
	-Digitaal fototoestel:	*OK (vakgroep kine)
	-Statief:	*Aankopen: Fnac?
	-Echo	*Dr. Peeters?

Vragenlijst 2 (p4 - 5) vertalen in het Frans zodat deze eventueel ook kan ingevuld worden door de patiënt zelf.

Patient's information and consent:

"Randomized trial comparing conventional vs short-course reduced volume conformal post-surgery radiation treatment in women with stage I or II breast cancer"

You have been referred for a post-operative radiotherapy of a breast tumour. We invite you to take part in a clinical study comparing a classic radiotherapy with a recent, new technique of irradiation (tomotherapy).

1. **Fundamental differences between a classic radiation treatment and an irradiation with tomotherapy:**

Classic radiation treatment:
With a classic radiotherapy the irradiation is delivered from 2 to 3 fixed positions. The treatment is delivered in 25 to 33 sessions over 5 to 7 weeks. Each session lasts about 10 minutes.
Advantages:
It is well known that this treatment considerably reduces the risk of local recurrence. Large studies with 20 to 30 years of follow-up have shown that ultimately this increases the chances of survival.
Disadvantages:
During the irradiation and in the following weeks most patients present temporarily various degrees of skin inflammation. Somewhat later complications in the longs and/or the heart, a lymphoedema (swelling of the arm) and a reduction of the mobility of the shoulder can sporadically occur. It is known that in the long term (20 – 30 years), irradiation can induce secondary tumours.

Experimental radiation treatment (tomotherapy):
This treatment system has been in use only since 4 years in the United States and since 3 years in Europe. The new equipment allows the radiation beams to be aimed from all angles, since the radiation beams turn around the patient. The radiation beams are also more precise and can be delivered more accurately.
The treatment is delivered in 15 sessions over 3 weeks. Each session lasts about 30 minutes.
Advantages:
The assumption is that the chances of cure are identical as compared to a classic radiation treatment. The new technique offers the potential advantage to reduce the previously mentioned complications by delivering less radiation doses to the surrounding normal tissues.
Disadvantages:
Considering the recent introduction of the equipment, there is not the same follow-up as with classic radiation treatment equipment. The skin toxicity could be for example more severe.

2. **What does your participation in this study imply?**

If you agree to take part in this study, you will be referred to one of the two treatments:

- either the classic radiation treatment
- or the “experimental” radiation treatment (tomotherapy).

The allocation to one of these two options will be done through a central computer system in the Universitair Ziekenhuis Brussel. The physician has no influence on the allocation.
The participation to the study is voluntary and can be interrupted at any time. Non-participation has no influence on the further treatment. If you take part in the study your family practitioner will be informed unless you refuse explicitly.
The medical ethics Committee might consult your medical file in order to control the good course of the study.
The data can be used for scientific reports or publications without releasing your identity. The results will be confidentially treated, in accordance with the Belgian Law protecting privacy.

Blood as well as tissue samples taken before or during the operation might be preserved for future research on breast cancer, but will not affect the treatment.
If you take part in this study complementary examinations will be carried out, before the treatment as well as 2 months after the end of the treatment and finally once a year during the 3 years following the treatment:

- Echocardiogram: This examination evaluates the hart valves and the pumping function. The examination moreover provides information about the velocity movements of the hart and blood flow.
- Respiratory function test: This examination measures how well the lungs work, and how efficient the exchange of oxygen and carbon dioxide is, within the lungs and the blood.
- Evaluation of the quality of life using a standardized European questionnaire (EORTC QLQ-C30).
- Clinical measurement of the circumference and mobility of the arms.
- Esthetical evaluation of the irradiated region: through photographs.

Medical Ethics Committee
This study has been approved by the Medical Ethics Committee of the UZ Brussel.

Insurance
Insurance has been contracted for this project according to the norms of the UZ Brussel (policy 45.145.223 clinical studies).

Contact person:
If you have any question and/or problem, you may always enquire the study coordinator:
Dr. Vincent Vinh-Hung
UZ Brussel
Radiotherapy Department
Tel: 02-477 60 41
GSM 0477- 788 900

I have read these notes and have taken good notice of the advantages, the possible disadvantages and the implications of the treatment. I have received answers to all my questions. Hereby I agree to take part in this study.

Date: Patient's signature:

Date: Doctor's signature:

Study coordinator: Dr. Vincent Vinh-Hung
Head of the Radiotherapy-Oncology Department: Prof. Dr. G. Storme

Information au patient et consentement éclairé: "Randomized trial comparing conventional vs short-course reduced volume conformal post-surgery radiation treatment in women with stage I or II breast cancer"

Vous avez été adressée pour recevoir une radiothérapie post-opératoire pour une tumeur du sein. Nous vous proposons de participer à une étude clinique qui compare une radiothérapie classique avec une récente, nouvelle technique de radiothérapie (tomothérapie).

1. Différences fondamentales entre un traitement de radiothérapie classique et une irradiation par tomothérapie:

Traitement par radiothérapie classique:
Avec un appareillage classique, les rayons sont délivrés à partir de 2 à 3 positions fixes.
Le traitement est effectué en 25 à 33 séances sur 5 à 7 semaines. Chaque séance se fait en une dizaine de minutes.
Avantages:
Il est bien connu que ce traitement apporte une nette diminution du risque de rechute locale. Des grandes études avec 20 à 30 ans de suivi ont montré que ceci apporte une amélioration des chances de survie.
Désavantages
Au cours des irradiations et dans les semaines qui suivent, la plupart des patientes présentent temporairement une inflammation cutanée plus ou moins importante. Un peu plus tardivement peuvent survenir sporadiquement des complications pulmonaires et/ou cardiaques, un lymphoedème (gonflement du bras) et une diminution de la mobilité de l'épaule. A long terme (20-30 ans), il est connu que les rayonnements ionisants peuvent induire des tumeurs secondaires.

Traitement par radiothérapie expérimentale (tomothérapie):
Cet appareil n'est utilisé que depuis 4 ans aux Etats Unis et depuis 3 ans en Europe. L'appareillage permet un rayonnement sous tous les angles, car ce sont les faisceaux de rayonnement qui tournent autour du patient. Les faisceaux de rayonnement sont aussi plus précis et peuvent être délivrés de manière plus délimitée.
Le traitement est effectué en 15 séances sur 3 semaines. Chaque séance se fait en une trentaine de minutes.
Avantages:
On présume que les chances de guérison restent identiques par rapport à un traitement de radiothérapie classique. La nouvelle technique offre l’avantage potentiel de réduire les complications précédemment décrites en délivrant moins de dose d’irradiation sur les tissus sains environnants.
Désavantages:
Etant donné l'introduction récente de l'appareil, on ne possède pas le même recul qu'avec la radiothérapie classique. La toxicité sur la peau pourrait par exemple être plus importante.

2. Quelles sont les implications si vous participez à l'étude ?

Si vous acceptez de participer à l'étude, vous serez adressée pour l’un des deux traitements:
- ou bien le traitement par radiothérapie classique
- ou bien le traitement par radiothérapie «expérimentale» (tomothérapie).

L’attribution pour l’une de ces deux options sera faite par un système informatique centralisé à l’Universitair Ziekenhuis Brussel. Le médecin traitant n’a pas d’influence sur l’attribution.
La participation à l’étude est volontaire et peut être interrompue à n'importe quel moment. La non-participation n’influence pas le traitement ultérieur. Si vous participez à l'étude, votre médecin de famille sera mis au courant sauf si vous le refusez explicitement.
Votre dossier médical peut être consulté par le Comité d’éthique médicale afin de contrôler le bon déroulement de l'étude.
Les données peuvent être utilisées dans des rapports scientifiques ou des publications sans que votre identité soit dévoilée. Les résultats seront traités de manière confidentielle en

accord avec la Loi belge sur la protection de la vie privée. Les prélèvements sanguins ou tissulaires effectués avant ou pendant l'opération peuvent être préservés pour des recherches futures sur le cancer du sein, mais n'influenceront pas le traitement.
En participant à l'étude vous aurez des examens complémentaires, avant le traitement et également 2 mois après la fin du traitement et finalement une fois par an pendant les 3 ans qui suivent le traitement:

- Echocardiogramme: Cet examen évalue la fonction des valves et de la pompe cardiaque. L'examen donne en outre une information sur la contractilité et le débit cardiaque.
- Epreuve fonctionnelle respiratoire: Cet examen mesure le bon fonctionnement des poumons, et mesure l'efficacité des échanges d'oxygène et de gaz carbonique, au niveau du poumon et du sang.
- Evaluation de la qualité de vie par un questionnaire standardisé Européen (EORTC QLQ-C30).
- Mesure clinique de la circonférence et de la mobilité des bras.
- Evaluation esthétique de la région irradiée: par des photographies.

Comité d'Ethique Médicale
Cette étude a été approuvée par le Comité d'Ethique Médicale de l'UZ Brussel.

Assurance
Une assurance a été prise pour ce projet suivant les normes de l'UZ Brussel (police 45.145.223 études cliniques).

Contact:
Si vous avez des questions et/ou des problèmes, vous pouvez toujours vous enquérir auprès du médecin coordinateur de l'étude:
Dr. Vincent Vinh-Hung
UZ Brussel
Service de Radiothérapie
Tel: 02-477 60 41
GSM 0477- 788 900

J'ai lu ces notes et bien noté les avantages, les désavantages éventuels et les implications du traitement. J'ai reçu les réponses à mes questions. J'accepte de participer à cette étude.

Date: Signature du patient:

Date: Signature du médecin:

Coordinateur de l'étude: Dr. Vincent Vinh-Hung
Chef du Département de Radiothérapie-Oncologie: Prof. Dr. G. Storme

Patiënteninformatie en toestemming:
"Randomized trial comparing conventional vs short-course reduced volume conformal post-surgery radiation treatment in women with stage I or II breast cancer"

U werd doorverwezen voor een post-operatieve radiotherapie omwille van een borsttumor. Wij stellen u voor om deel te nemen aan een studie die een klassieke radiotherapie vergelijkt met een recente, nieuwe bestralingstechniek (tomotherapie).

1. **De fundamentele verschillen tussen een klassieke radiotherapeutische behandeling en de bestraling met tomotherapie:**

Een klassieke radiotherapeutische behandeling:
Met het klassieke apparaat worden de stralen vanuit 2 à 3 vaste invalshoeken toegediend.
De behandeling bestaat uit 25 tot 33 zittingen die over 5 à 7 weken worden toegediend. Elke bestraling neemt een 10-tal minuten in beslag.
Voordelen:
Van deze behandeling is goed gekend dat ze het risico op een lokaal herval beduidend verkleind. Grote studies met 20 tot 30 jaar ervaring hebben aangetoond dat dit uiteindelijk de overlevingskans vergroot.
Nadelen:
Tijdens de bestralingen en de weken nadien vertonen de meeste patiënten in min of meerdere mate tijdelijke huidontsteking. Op iets langere termijn kunnen sporadisch long- en/of hart complicaties, lymfoedeem (zwelling van de arm) en vermindering van de schoudermobiliteit optreden. Van ioniserende straling is gekend dat ze op lange termijn (20-30 jaar) secondaire tumoren kan induceren.

De experimentele radiotherapeutische behandeling (tomotherapie):
Dit apparaat is pas sinds 4 jaar in de Verenigde Staten en sinds 3 jaar in Europa in gebruik. Met het nieuwe toestel kunnen de stralen vanuit alle hoeken gebruikt worden aangezien, de stralingsbundel rond de patiënt draait. Deze bestralingsbundel is ook preciezer en kan nauwkeuriger worden toegediend.
De behandeling bestaat uit 15 zittingen die over 3 weken worden toegediend. Elke bestraling neemt een 30-tal minuten in beslag.,
Voordelen:
De veronderstelling is dat de genezingskansen identiek zijn vergeleken met een klassieke radiotherapeutische behandeling. De nieuwe techniek biedt het potentiële voordeel voornoemde complicaties te verminderen door minder stralingsdosis te geven op de omgevende normale weefsels.
Nadelen:
Gezien de recente invoering van het apparaat, bezit men niet over dezelfde ervaring als met het klassieke radiotherapeutisch apparaat. De huidtoxiciteit zou b.v. ernstiger kunnen zijn.

2. **Wat betekent het als U deel zou nemen aan de studie?**

Indien U toestemt om aan de studie deel te nemen, zal u naar een van beide behandelingen doorverwezen worden voor:
- ofwel de klassieke radiotherapeutische behandeling
- ofwel de “experimentele” radiotherapeutische behandeling (tomotherapie).

De toewijzing naar één van deze twee opties zal door een gecentraliseerd computersysteem in het Universitair Ziekenhuis Brussel worden gedaan. De behandelende arts heeft op deze toewijzing geen invloed.
De deelname aan deze studie is vrijwillig en kan op elk moment onderbroken worden. Niet-deelname heeft geen invloed op de verdere behandeling. Indien u deel neemt aan de studie zal uw huisarts op de hoogte gebracht worden tenzij u dit uitdrukkelijk weigert.
Uw medisch dossier kan door het geneeskundige Commissie voor ethiek geraadpleegd worden teneinde het goede verloop van de studie te controleren.

De gegevens kunnen tijdens wetenschappelijke rapporten of publicaties gebruikt worden zonder uw identiteit weer te geven. De resultaten zullen vertrouwelijk worden behandeld, in overeenstemming met de Belgische Wetgeving op de bescheming van de persoonlijke levenssfeer. Bloed evenals weefselmateriaal dat voor of gedurende de operatie gepreleveerd werd, kan bewaard worden voor toekomstige onderzoeken voor borst kanker, maar zullen geen verder invloed hebben op de behandeling.
Door aan de studie deel te nemen zult u aanvullende onderzoeken ondergaan die, vòòr de behandeling en eveneens 2 maanden na het einde van de behandeling en tenslotte eens per jaar gedurende 3 jaar na de behandeling verricht zullen worden:

- Echocardiogram: Dit onderzoek bepaalt hoe de functie van de hartkleppen en de pompfunctie is. Het onderzoek geeft bovendien informatie over de snelheid van het bewegen van het hart en de bloedstroom.
- Longfunctieonderzoek : Dit onderzoek meet hoe goed de longen werken, en hoe efficiënt de wisselwerking tussen zuurstof en kooldioxide is, binnen de longen en het bloed.
- Evaluatie van de levenskwaliteit door een gestandaardiseerde Europese vragenlijst (EORTC QLQ-C30).
- Klinische meting van de omtrek en beweegbaarheid van de armen.
- Esthetische evaluatie van de bestraalde regio: door middel van foto's.

Commissie Medische Ethiek
Deze studie werd goedgekeurd door de Commissie Medische Ethiek van het UZ Brussel.
Verzekering
Voor dit project werd een verzekering afgesloten volgens de normen van het UZ Brussel (polis 45.145.223 klinische studies)
Contact persoon:
Bij vragen en/of problemen kan U steeds terecht bij de studie coördinator:
Dr. Vincent Vinh-Hung
UZ Brussel
Dienst Radiotherapie
Tel: 02-477 60 41
GSM 0477- 788 900

Ik heb deze notitie doorgelezen en goed nota genomen van de voordelen, de eventuele nadelen en de gevolgen van de behandeling. Ik heb op alle vragen antwoord gekregen.
Ik stem hierbij toe om deel te nemen aan deze studie.

Datum: Handtekening Patiënt:

Datum: Handtekening arts:

Studie coördinator: Dr. Vincent Vinh-Hung
Diensthoofd Radiotherapie-Oncologie: Prof. Dr. G. Storme

PROTOCOL BORST: Dr VINH

Alle patiënten met een mastectomie, die in aanmerking komen voor een vergelijkende studie (dr Vinh) en behandeld worden op linac 5 (randomisatie + informed consent) moeten in ruglig bestraald worden. Patiënten worden gerandomiseerd (behandeling op L3 of op L5) door Sven of Marleen. Myriam of Mark zorgen voor het toewijzen van de patiënten.

HOUDING + MARKERINGEN

RUGLIG:

HULPMIDDELEN: licht blauw (orfit) steun

- Thorax kss a00
- Lat steun a01
- Armsteun a03
- Kniesteun a04
- Plaat a05

neksteun (+ flap); A,B, of C
eventueel nekspie
voetensteun

HOUDING: patiënt moet steeds recht voor zich kijken

armen boven hoofd

patiënt moet steeds met dezelfde hand (contralaterale kant van mastectomie) linker of rechter pols vastnemen

MARKERINGEN: anterieur; lengtelaser in het midden van het sternum
zijdelingse laser onder het sternum (processus xiphoideus)
lateraal; laterale markeringen (kruis) in het midden van de thorax, bilateraal

NOOT: zorg ervoor dat de lengtelaser in het midden van de neus, de kin, de incisura jugularis, het sternum, het voetenkussen en tussen de beide benen loopt

Alle patiënten met een tumorectomie, die in aanmerking komen voor een vergelijkende studie (dr Vinh) en behandeld worden op linac 5 (randomisatie + informed consent) worden in ruglig of, indien mogelijk, in buiklig bestraald. Patiënten ondergaan dan een simulatie in buiklig en ruglig omdat ze nadien in dezelfde houdingen moeten gescand worden (radiologie). De uiteindelijke houding wordt bepaald na planning! Patiënten worden gerandomiseerd (behandeling op L3 of op L5) door Sven of Marleen. Myriam of Mark zorgen voor het toewijzen van de patiënten.

HOUDING + MARKERINGEN

RUGLIG:

Zie mastectomie

BUIKLIG:

HULPMIDDELEN: gele (orfit) steun

staaf a43 in C (aio plaat)

liefst geen hoofdkussen

voetensteun (+ eventueel 1 of 2 gele kussens)

HOUDING: buiklig

armen boven hoofd

patiënt neemt met beide handen de staaf (a43) vast

hoofd naar links of rechts

Let erop dat de bestraalde borst voldoende vrij hangt (midden van het sternum op uitsparing gele steun)
Plaats de koolstofplaat aan de contralaterale kant

MARKERINGEN: posterieur; lengtelaser in het midden van de rug
zijdelingse markering van de bovenkant en onderkant van de uitsparing, bilateraal!!!
lateraal; laterale markeringen (hoogtelijn) in het midden van de buik, bilateraal

NOOT: zorg ervoor dat de lengtelaser in het midden van het hoofd, de rug, het voetenkussen en tussen de beide benen loopt.

RE: CTV boost after BCS

VINH HUNG Vincent

Sent: Saturday, April 02, 2011 10:14 PM

To: Guy Soete [Guy.Soete@uzbrussel.be]

Cc: Nele.Platteaux@uzbrussel.be; Nicolas.Christian@uzbrussel.be; Benedikt Engels [benedikt.engels@gmail.com]; Christine.Collen@uzbrussel.be; Dirk.VanDenBerge@uzbrussel.be; Geertje Miedema [Geertje.Miedema@uzbrussel.be]; Hilde.VanParijs@uzbrussel.be; Mark.DeRidder@uzbrussel.be; Mia.Voordeckers@uzbrussel.be; Simin Sadeghi [Simin.Sadeghi@uzbrussel.be]

Beste Guy,

Sorry, ik ben net terug van SASRO en andere.

Inderdaad recent stelt van Mourik een expansie van 15 mm « minus tumour-free resection margin » rond excisie holte,
http://www.ncbi.nlm.nih.gov/pubmed/20199818
zoals NSABP-B39, Kirby, etc…
http://www.ncbi.nlm.nih.gov/pubmed/20080310

Maar oplet: deze CTV expansie van 10 mm of 15 mm geld enkel voor PBI Partial Breast Irradiation.
Bij Whole breast radiotherapy WBRT, lijkt deze marge gevaarlijk.
Bvb met resectie holte van 5 cm diameter, +15 mm CTV marge, +5 mm PTV marge, wordt volume van 65 cc à 382 cc, meer dan helft van een gemiddeld borst.

Voor de Tomoborst studie, CTV expansie was gebaseerd op gegevens van Vicini:
http://www.ncbi.nlm.nih.gov/pubmed/15465188
Vicini stelde dat 10 mm marge voldoende is voor PBI partiele borst bestraling.
Maar bij WBRT volledig borst RT, voor boost reken houd ik van de hoogst risico worst-case scenario dat een resectie marge rakelings de tumor, reken houdend dat "Among the 121 reexcision specimens with residual invasive carcinoma… In these specimens, the mean and median values of the maximum extension of invasive carcinoma beyond the edge of the initial excision site were 6.4 mm and 5.0 mm, respectively (range, 1.0 –20.0 mm; SD, 4.9 mm)", reken houdend dat Vicini's reeks van patienten een bijzonder hoge risico groep was (impliciet bij reexcisie), dus CTV van 7 mm, 7 mm is groter dan de gemidelde en ruim meer dan 50% van de worst-case high-risk.

Met vele groeten,

Vincent

De : Guy Soete [mailto:Guy.Soete@uzbrussel.be]
Envoyé : jeudi, 24. mars 2011 10:09
À : VINH HUNG Vincent
Cc : Nele.Platteaux@uzbrussel.be; Nicolas.Christian@uzbrussel.be; Benedikt Engels; Christine.Collen@uzbrussel.be; Dirk.VanDenBerge@uzbrussel.be; Geertje Miedema; Hilde.VanParijs@uzbrussel.be; Mark.DeRidder@uzbrussel.be; Mia.Voordeckers@uzbrussel.be; Simin Sadeghi
Objet : CTV boost after BCS

Beste Vincent,

Hopelijk alles goed met u en de familie!
Mag ik even je advies?
Er is al enige tijd discussie aan de gang over de marges van tumorectomie-regio naar CTV voor boost na BCS.
Literatuur stelt >=10mm voor; EORTC zelfs 15mm.
In uw protocol van 2007 wordt 7mm gevraagd.
De 7mm lijkt me persoonlijk realistischer, **maar hoe kunnen we dit motiveren**?
Beste groeten vanuit onze nieuwe site te ASZ Aalst,

Guy
